# Cortical and subcortical functional specificity associated with response inhibition

**DOI:** 10.1016/j.neuroimage.2020.117110

**Published:** 2020-10-15

**Authors:** Leah Maizey, C. John Evans, Nils Muhlert, Frederick Verbruggen, Christopher D. Chambers, Christopher P.G. Allen

**Affiliations:** a Cardiff University Brain Research Imaging Centre, School of Psychology, Cardiff University, United Kingdom; b Division of Neuroscience and Experimental Psychology, University of Manchester, United Kingdom; c Department of Experimental Psychology, Ghent University, Belgium

**Keywords:** Response inhibition, Response execution, Action control, Action updating, Basal ganglia, Action pathways, pre-SMA, pre-supplementary motor area, IFG, inferior frontal gyrus, BG, basal ganglia, STR, striatum, GPe, globus pallidus externa, GPi, globus pallidus interna, SN, substantia nigra, STN, subthalamic nucleus, THAL, thalamus, ROI, region of interest, %BOLD, percent change in blood oxygen level dependent signal, SST, stop-signal task, DT, double-reponse task, IT, ignore task, fMRI, functional magnetic resonance imaging, GLM, general linear model, BF, Bayes Factor, RT, reaction time

## Abstract

Is motor response inhibition supported by a specialised neuronal inhibitory control mechanism, or by a more general system of action updating? This pre-registered study employed a context-cueing paradigm requiring both inhibitory and non-inhibitory action updating in combination with functional magnetic resonance imaging to test the specificity of responses under different updating conditions, including the cancellation of actions. Cortical regions of activity were found to be common to multiple forms of action updating. However, functional specificity during response inhibition was observed in the anterior right inferior frontal gyrus. In addition, fronto-subcortical activity was explored using a novel contrast method. These exploratory results indicate that the specificity for response inhibition observed in right prefrontal cortex continued downstream and was observed in right hemisphere subcortical activity, while left hemisphere activity was associated with right-hand response execution. Overall, our findings reveal both common and distinct correlates of response inhibition in prefrontal cortex, with exploratory analyses supporting putative models of subcortical pathways and extending them through the demonstration of lateralisation.

## Highlights

• Anterior specificity in cortical activity to response inhibition was identified.
• Methodological development of a compound contrast methodology optimising data usage.
• Exploratory demonstration of lateralised subcortical action control pathways.

## 1 Introduction

Response inhibition, the ability to suppress motor responses that are inappropriate or no longer required, supports flexible, goal-directed behaviour. Studies have repeatedly indicated that, neuroanatomically, the right inferior frontal gyrus (IFG) and the pre-supplementary motor area (pre-SMA) are crucial in motor inhibition (e.g. Aron et al., 2003; Nachev et al., 2007). However, there is also the possibility that comparable functional activity may be observed during other forms of control, such as action updating that does not involve the cancellation of responses (e.g. Chatham et al., 2012; Dodds et al., 2011; Erika-Florence et al., 2014; Hampshire, 2015; Hampshire et al., 2010). As such, the extent to which the right IFG, pre-SMA and associated regions are specialised in their role in response inhibition is ambiguous.

**Fig. 1:**
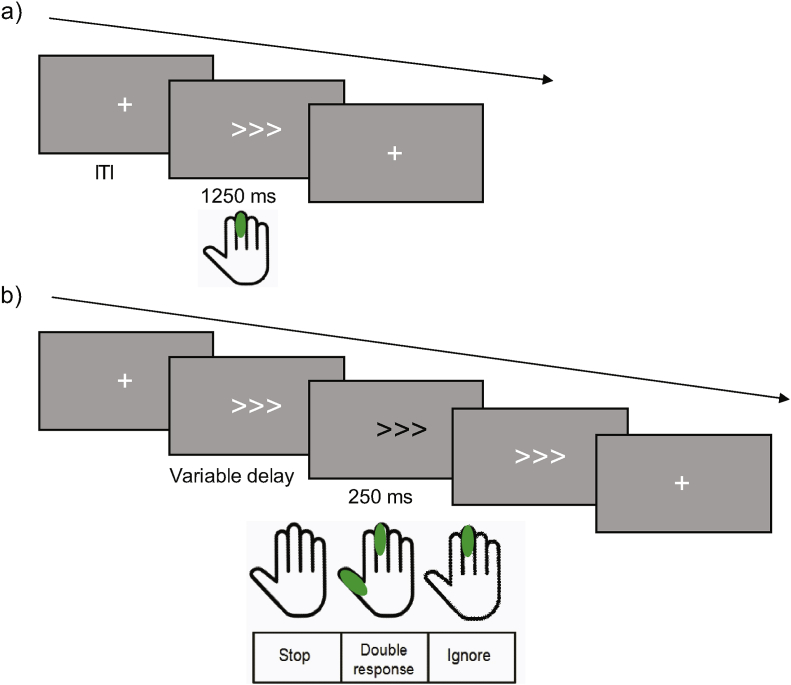
**The context-cueing paradigm**. (a) Participants were instructed to respond to the direction of white arrows (go or no-signal trials) as fast and as accurately as possible using their right index or middle fingers. (b) Signal trials (the white arrow turning black after a variable delay) were presented on 33% of trials. Task context was indicated at the start of each block. In the stop context, participants were instructed to withhold their response upon presentation of a signal. In the double-response context, participants were instructed to execute an additional right thumb response when the signal appeared. In the ignore context, participants were instructed to ignore the presence of the signal and to respond as if no signal were presented. Fixation crosses were presented prior to each trial for the duration of the inter-trial interval (ITI), which was adjusted between 500 ​ms, 1000 ​ms and 2000 ​ms. The delay between stimulus and signal onset was variable and titrated to individuals stop-signal performance to achieve successful response inhibition of ~50% on stop-signal trials. Delays were randomised within contexts, but equivalent across contexts.

One commonly used task to measure response inhibition is the stop-signal task (SST; Logan and Cowan, 1984; Verbruggen et al., 2019; Verbruggen and Logan, 2008). During the SST, participants execute a motor response to a stimulus on the majority of trials but are required to occasionally stop the response upon presentation of an infrequent, yet salient, stop signal. The specificity of neurocognitive systems for response inhibition can be tested by comparing behaviour or brain activity in the SST with control tasks in which actions are updated without response inhibition. One such task is the double-response task (DT) in which stimulus presentation mimics the SST but requires the execution of an additional rapid response following the infrequent signal, as opposed to the inhibition of a response (Chatham et al., 2012; Dodds et al., 2011; Erika-Florence et al., 2014; Hampshire, 2015; Hampshire et al., 2010; Tabu et al., 2011; Verbruggen et al., 2010). Perceptual confounds are also controlled through the introduction of an additional task in which participants are instructed to ignore the infrequent signal (the ignore task, IT). Collectively, these three tasks – SST, DT and IT – comprise the context-cueing paradigm (Verbruggen et al., 2010; Verbruggen and Logan, 2009) employed here (Fig. 1). The use of the context-cueing paradigm in conjunction with functional magnetic resonance imaging (fMRI) allowed us to explore the specificity of neuronal activity across different action updating conditions.

**Fig. 2:**
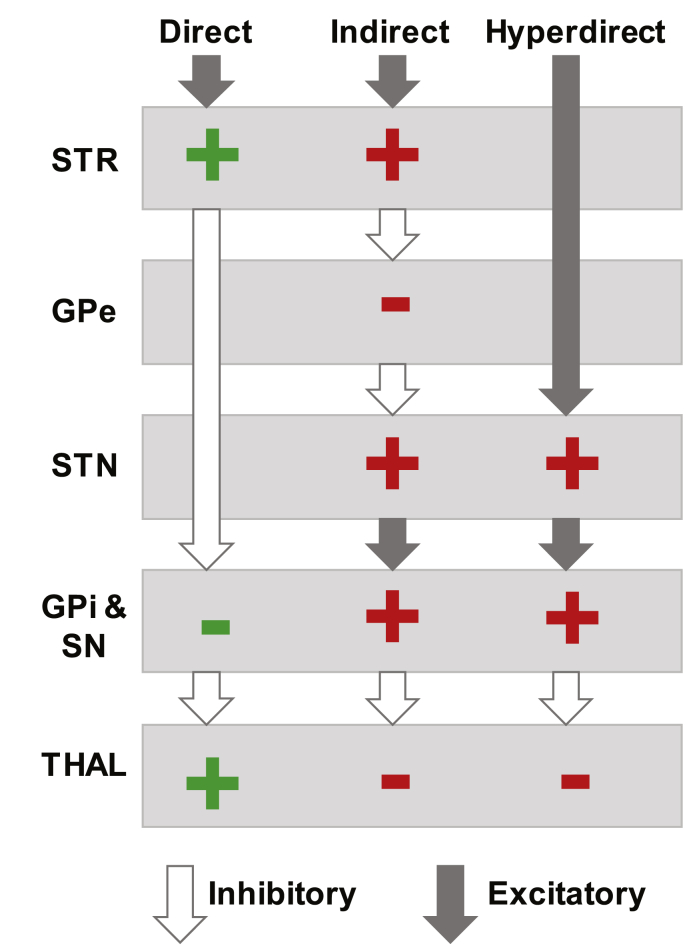
**Subcortical pathways**. Subcortical pathways model of response execution and response inhibition (Albin et al., 1989; Alexander and Crutcher, 1990; DeLong, 1990; Nambu et al., 2002). Filled arrows represent excitatory projections, and unfilled arrows represent inhibitory projections. ​+ ​symbols indicate up-regulation of neuronal activity and – symbols indicate down-regulation of neuronal activity within specified structures under the activation of each pathway. The *direct* pathway (green symbols) is theorised to enable responses to be executed. The *indirect* and *hyperdirect* pathways (red symbols) are proposed to inhibit the execution of actions. STR ​= ​striatum, GPe ​= ​globus pallidus externa, STN ​= ​subthalamic nucleus, GPi ​= ​globus pallidus interna, SN ​= ​substantia nigra, THAL ​= ​thalamus.

The context-cuing paradigm and fMRI allows us to explore another key set of regions associated with control; the basal ganglia (BG) and thalamic nuclei. Converging neuroanatomical evidence suggests that the IFG and pre-SMA exert influence over subcortical activity in the control of actions, including response inhibition (e.g. Aron et al., 2007; Aron and Poldrack, 2006; Jahfari et al., 2011; Rae et al., 2015). Further, efficiency of inhibitory control is dependent on the strength of fronto-basal ganglia connectivity (Chavan et al., 2017; Forstmann et al., 2012; Jahfari et al., 2012; Matar et al., 2019). Motor output through the thalamus (THAL) is thought to be under the direction of signals originating from frontal regions via three pathways through the basal ganglia; the direct, indirect and hyperdirect pathways (see Fig. 2; Albin et al., 1989; Alexander and Crutcher, 1990; DeLong, 1990; Nambu et al., 2002). When executing a response, the fronto-striatal-pallidal (direct) pathway is activated. Inhibition of actions is suggested to operate via the indirect and hyperdirect pathways, acting through the subthalamic nucleus (STN), substantia nigra (SN) and globus pallidus interna (GPi) to suppress THAL output and to block direct activation. The hyperdirect pathway is so-called because it innervates the STN directly, resulting in fast inhibition (Nambu et al., 2002). This pathway has been linked with ‘reactive’ inhibition, the active suppression of all (global) responses (Aron, 2011, 2008; Aron et al., 2016; Aron and Poldrack, 2006; Verbruggen et al., 2019; Wessel et al., 2019, 2016). In comparison, the indirect route involves projections to the striatum (STR) and the external segment of the globus pallidus (GPe) before reaching the STN to suppress thalamico-cortico output (Albin et al., 1989; Alexander and Crutcher, 1990). Among other functions, this slower route is theorised to provide tonic suppression in anticipation of withholding of actions (i.e. proactive inhibition; e.g. Aron, 2011, 2008; Majid et al., 2013; Zandbelt et al., 2013). Although there is some controversy over the specificity of the pathways in terms of behavioural inhibition (e.g. Aron et al., 2014a, Aron et al., 2014; Hampshire and Sharp, 2015; see also Meyer and Bucci, 2016), and more fundamentally their existence, there is also the question of how (and if) these pathways are detectable using fMRI.

The primary pre-registered goal of the current work was to establish the neuroanatomical distribution of inhibitory and non-inhibitory action updating, in terms of their specificity and overlap (see https://osf.io/zqefx/, section 3). Our pre-registered hypotheses were that both forms of action updating would be associated with widespread fronto-parietal activity, corresponding to commonalities of control. However, we also expected specificity with more right-lateralised activity associated with response inhibition compared to non-inhibitory action updating, and more left-lateralised activity associated with non-inhibitory action updating compared to inhibitory action updating. We expected right IFG and pre-SMA activity to be common to both SST and DT but anticipated differences in their degree of recruitment. We further hypothesised that the observed pattern of subcortical activity would be consistent with the putative action control pathways. For example, when actions are inhibited we expected to observe changes in STN, SN, GPi and THAL activity corresponding to the hyperdirect pathway (Fig. 2). While hypotheses with respect to subcortical activity were pre-registered the corresponding analyses methods were not and therefore related analyses should be considered exploratory.

The main hypotheses and methods for this study were pre-registered prior to data collection (https://osf.io/zqefx/ and https://osf.io/27gmh/).

## 2 Materials and methods

### 2.1 Participants

Thirty right-handed participants (5 males), aged 18–29 years (*M* ​= ​21.43, *SD* ​= ​2.64), were included in the study. All had normal or corrected-to-normal vision, were neurologically healthy and screened for contraindications to MRI. Informed consent was received from each participant and all methods were approved by the ethics committee at the School of Psychology, Cardiff University. Participants were reimbursed at a rate of £10 per hour for their time.

### 2.2 Protocol pre-registration

The study protocol was pre-registered prior to data collection, see here: https://osf.io/zqefx/ and here: https://osf.io/27gmh/. Deviations were made in relation to how the subcortical regions of interest were selected. These decisions were made prior to data analyses and are fully detailed in the supplementary information (SI 1.5).

### 2.3 Task design

The task design is outlined in Fig. 1. For all conditions, participants were instructed to respond to the direction of a central white arrow (1250 ​ms, irrespective of whether a response was made) with either their right index or right middle fingers. Signals (i.e. the white arrow turning black for 250 ​ms), were presented on 33% of trials. Participants were required to respond to these signals in accordance with a cue preceding each task block (7000 ​ms). In the SST (cue: STOP), participants were required to withhold their response and to not respond to the direction of the arrow. In the DT (cue: DOUBLE), participants were required to execute an additional thumb response after responding to the direction of the arrow. In the IT (cue: IGNORE), participants were required to ignore the presence of the signal and respond to direction of the arrow. Participants were instructed to execute responses as fast and as accurately as possible where required and to not slow their responses in order to perform better on signal trials. The delay between the stimulus and signal onsets was initially based on psychophysical inhibition functions established during training, matched across contexts and adjusted throughout the scan session (for a detailed description, see sections 4.2.3 and 5.3 here: https://osf.io/zqefx/ and a clarification point 1 here: https://osf.io/27gmh/). Signal presentation was pseudo-randomised so that a maximum of three signals were presented in succession. Fixation crosses appeared during the inter-trial interval (ITI) for either 500 ​ms, 1000 ​ms or 2000 ​ms to reduce automation of participants’ responses. Arrow direction and ITIs were randomised but occurred with equal probability in each block. Further technical detail is reported in SI 1.2.

### 2.4 fMRI protocol

A 3T GE GDx scanner, equipped with an 8-channel head coil was used. Whole-brain functional images were acquired using an echo planar imaging (EPI) sequence with AC-PC alignment (TR ​= ​3000 ​ms, TE ​= ​35 ​ms, matrix size: 64 ​× ​64, flip angle: 90° in-plane resolution: 3.4 ​mm ​× ​3.4 ​mm, 3.4 ​mm slice, no gap). Interleaved slices were acquired in an ascending direction. 156 ​vol were acquired over the course of each run (1248 ​vol in total), such that a single fMRI run covered the duration of each of the behavioural runs. Each run was preceded by the acquisition of 4 dummy scans. A T1-weighted anatomical scan (172 slices; voxel resolution: 1 ​× ​1 ​× ​1 ​mm; TR: 8 ​ms; TE: 3 ​ms, inversion time: 450 ​ms, flip angle of 20°, matrix size: 256 ​× ​256) and 2 field maps (3D spoiled, gradient-recalled echo sequence, TR ​= ​20 ​ms, TE ​= ​7 and 9 ​ms) were acquired after EPIs for each participant. In addition, to localise hypointense STN (Manova et al., 2009) a susceptibility weighted image (SWI; 3D spoiled gradient recalled echo sequence, TR ​= ​57 ​ms, TE ​= ​39 ​ms, 2 ​mm isotropic resolution) was also acquired in the coronal and axial planes for each participant.

### 2.5 Study procedure

Prior to the fMRI session all participants completed an initial training session (see section 4.2.3 here: https://osf.io/zqefx/). Before testing, participants completed one block of each task, presented in a randomised order, to remind them of the task instructions. This data was not saved or analysed. The testing session included 8 fMRI runs of the behavioural task. Each task block consisted of 18 trials (6 signals) and the context pseudo-randomly switched every block, so that no context was repeated across successive blocks. For each fMRI run, 3 blocks of each task were presented, and each block was separated by task context cues. As such, 432 trials per task (144 signals) were presented in each testing session. The delays between stimulus and signal onsets were adjusted after completion of every 2nd fMRI run to ensure successful inhibition on stop signal trials remained at ~50%. If performance on any of the tasks fell beyond pre-registered benchmarks, standardised feedback was provided (see sections 4.2.3 and 5.3 here: https://osf.io/zqefx/). Throughout testing, physiological measures of cardiac and respiration rate, O_2_ troughs and end-tidal CO_2_ were taken. Full details are reported in SI 1.3.

### 2.6 Statistical analyses

All frequentist analyses were conducted in SPSS (IBM Corp (2015), version 23). The Holm-Bonferroni method (Aickin and Gensler, 1996) was used to correct for multiple comparisons and is shown as the *p*-value subscript where relevant (exploratory analyses were uncorrected). Bayesian equivalents of all frequentist tests were computed using either the ‘default’ prior settings in JASP (JASP Team (2019), version 0.9.2.0) or using the JZS ‘default’ prior with a scale factor of r ​= ​√$\frac{1}{2}$ (Rouder et al., 2009; Wetzels and Wagenmakers, 2012) in custom-written Matlab scripts (Mathworks (2015); this applies to all exploratory analyses with the exception of repeated measures ANOVA which were computed in JASP; outputs can be found here: https://osf.io/g4chs/). For repeated measures ANOVA in JASP, Bayes Factors (BFs) were taken for the model compared to the null (i.e. BF_10_). For interaction effects, BF_10_ of the model with the interaction was compared against the BF_10_ of the model with only the main effects (i.e. BF_10_ for the full model ​+ ​interaction/BF_10_ for the full model without the interaction; Mathôt, 2017). BFs were interpreted as follows: (1) BF ​> ​3 suggests ‘substantial evidence’ for H1 relative to H0, (2) BF of ~1 suggests limited sensitivity of the experiment to detect effects, and (3) BF ​< ​1/3, provides ‘substantial evidence’ for H0 relative to H1 (Jeffreys, 1961).

### 2.7 fMRI analyses

#### 2.7.1 Pre-processing

In-house scripts were used to remove physiological regressors from the EPI data prior to pre-processing (Bright and Murphy, 2013). Subsequent pre-processing and analyses were carried out using FEAT (v. 5.98) in FSL 4.1.4. (FMRIB, Oxford, UK; Jenkinson et al., 2012; Smith et al., 2004; Woolrich et al., 2009). EPI data were motion-corrected, subjected to field map based correction (B0 unwarping^1^), slice time corrected, spatially smoothed using a 5 ​mm full-width-half-maximum Gaussian kernel, temporally high-pass filtered at 128 ​s and pre-whitened. The resulting images were entered into a general linear model (GLM) and events modelled after convolution with a canonical hemodynamic response function, with temporal derivatives taken into account. For all analyses, events included signal and no-signals across the SST, DT and IT. For exploratory analyses, additional events included correct stop signal, incorrect stop signal and fixation crosses across these tasks. All contrasts conducted to meet the primary, secondary and exploratory aims are referenced in the supplementary information (see SI sections 2.2, 2.3, respectively).

#### 2.7.2 Whole-brain analyses

Whole brain cluster-based analyses were conducted with Z ​> ​2.3 and *p* ​< ​0.05, using Gaussian Random Field theory (Friston et al., 1991). At the individual level, fixed analyses were conducted. Mixed-effects analysis was used at the group level and all imaging data registered to a 2 ​mm Montreal Neurological Institute (MNI) template brain, using a 12 degree-of-freedom linear registration. All analyses were explored in MNI space. Analyses used to determine brain-behaviour relationships are reported in SI 2.3. All pre-registered contrast, conjunction (Nichols et al., 2005) and disjunction analyses are outlined in SI 2.2.

#### 2.7.3 Region of interest analyses

Regions of interest (ROIs) were created for all cortical and subcortical structures of interest. IFG ROIs were defined as the combination of the *pars opercularis* and *pars triangularis* as specified by the Harvard-Oxford cortical atlas (Desikan et al., 2006; Frazier et al., 2005; Goldstein et al., 2007; Makris et al., 2006). For analyses specifically related to the *pars opercularis* and *pars triangularis* overlapping voxels were removed from respective masks. Pre-SMA was identified as the SMA region for where y ​> ​0 in the Automated Anatomical Labelling atlas (Aron and Poldrack, 2006; Tabu et al., 2011). THAL was defined as the region specified by the Harvard-Oxford subcortical atlas (Desikan et al., 2006; Frazier et al., 2005; Goldstein et al., 2007; Makris et al., 2006). STN were manually identified by 2 authors (LM and NM) from the SWI images (the reliability of identification between authors was good: ICC ​= ​0.707; *r*_(28)_ ​= ​0.626, *p* ​< ​0.001). Only regions identified by both as the STN were included in the final masks. All other ROIs were specified by the Atlas of the Basal Ganglia (ATAG; (Keuken et al., 2014); deviation from pre-registered protocol, see SI 1.5). All ROIs were linearly transformed to 2 ​mm MNI space using FLIRT (Jenkinson et al., 2002; Jenkinson and Smith, 2001) in FSL, threshold to 25% (50% for the pre-SMA) and binarised using FSLMATHS. Overlapping voxels between regions were excluded from all analyses, however, those identified as STN were maintained in favour of overlap with SN. Percent blood oxygen level dependent signal change (%BOLD) was extracted from each ROI for signal ​> ​no-signal specified contrasts using FeatQuery.

#### 2.7.4 Exploratory pathways analysis

These analyses focused on the correspondence between the pattern of observed BOLD activations and those expected due to the functionally specific activity of the putative action control pathways. Analyses were conducted to assess: 1) the pattern of activations in cortical and subcortical ROIs, and 2) whether these patterns were consistent with the putative pathways. Across the neuroimaging literature a variety of contrasts are often used to refer to similar states of interest (e.g. stop signal ​> ​stop no-signal (go) and stop signal ​> ​null (implicit baseline) have both been used to demonstrate activity associated with response inhibition; Aron et al., 2007; Aron and Poldrack, 2006). The choice of contrasts can appear arbitrary and offer opportunities for researcher degrees of freedom. To avoid this, we developed an approach that incorporates all reasonable contrasts that have potential to inform the hypotheses. Data included 131 separate contrasts (see SI 1.6), each entered into a separate GLM, where second level FEAT analysis was applied across runs for each participant. Separate GLMs were used to optimise the contribution of the signal for each contrast (alternative processing strategies and considerations are described here: https://osf.io/zkq7h/). Contrasts were divided into those pertaining to response execution and response inhibition, and then further subdivided into contrasts representative of proactive (preparatory) inhibition and reactive inhibition (the active stopping of an action in response to the signal). Here response execution related contrasts were expected to result in activation patterns consistent with the direct pathway, and response inhibition contrasts were expected to result in activity conforming to the indirect and hyperdirect pathways, where the indirect pathway might link to proactive contrasts and the hyperdirect ROIs might be expressed more clearly by reactive contrasts, following their putative role (Nambu et al., 2002). These contrasts were allocated initially blindly and then by consensus by three of the authors (see SI 1.6). %BOLD signal change was drawn from each ROI for each participant, and means were calculated across each compound contrast set for use in subsequent analyses (ANOVA etc. see below). This procedure produced point value estimates for each participant and ROI under each of the three conditions; action execution, action inhibition (pro-active) and action inhibition (reactive). As differences between contrasts, within a condition set, are not of interest (e.g. different baselines such as fixation and ignore events) but the similarities are (e.g. stop signal present in all contrasts), the averaging process should theoretically result in variables that are influenced predominantly by the commonalities, enhancing statistical reliability and the stability of related inferences.

Initial analysis applied a repeated measures ANOVA to the compound data with factors of site (16 levels of lateralised cortical and subcortical ROIs) and condition (response execution vs. response inhibition). Single sample t-tests were then applied to each ROI under each condition. Interrelationships between activated regions was assessed with a series of partial (moderator/mediator) analyses (Baron and Kenny, 1986; Judd et al., 2001). Here, any two regions indicating evidence for activation (i.e. result from one-sample t-tests ​= ​*p*<0.05 or BF>3, separately), were regressed upon one another. The linear model of these regressions include intercept coefficients, derived as t-statistics. These statistics were representative of one region being active, relative to mean zero, when the covariant was taken into account. *p*-values and BFs were then derived.

The second set of analyses made use of the compound contrast data sets applied to lateralised subcortical regions. To assess the consistency between the pattern of activity observed and that expected according to the pathways descriptions, we applied models representative of each pathway to the data. A BF was derived for each pathway under their behaviourally relevant condition (e.g. %BOLD from contrasts reflective of response inhibition from ROIs comprising the indirect pathway). This involved taking the BFs based on the single sample *t*-test (as described above), for each region proposed to be involved in each pathway, separately. For all subcortical regions not proposed to be involved, the inverse of the BF was taken as representative of evidence of zero activity. Then, taking advantage of BFs being transitive, the product of these was taken as evidence of the consistency between activity of the proposed pathway and observed activity under behaviourally relevant conditions. For example, the product BF for the hyperdirect pathway was the result of BFs from right GPi, right STN, right SN and right THAL (i.e. regions proposed to contribute to the pathway) and inverse BFs from the right GPe, right STR and all left subcortical structures (i.e. regions not proposed to contribute to the pathway). By applying this to the corresponding compound contrasts we were able to assess the weight of evidence for each pathway under their behaviourally relevant conditions (e.g. direct pathway with response execution contrasts). Finally, to confirm that any correspondence was not the result of over-fitting, the analyses were applied to the behaviourally inappropriate compound data (e.g. %BOLD from response execution contrasts applied to the indirect (inhibitory) pathway). Alternative modelling approaches are reported here: https://osf.io/zkq7h/.

## 3 Results

We first describe the outcome of pre-registered behavioural analyses and our primary imaging analyses aimed at delineating the pattern of cortical activity under inhibitory and non-inhibitory action updating conditions. These are followed by analyses aimed at exploring the expression of the putative subcortical pathways. Additional pre-registered brain-behaviour analyses are reported in SI 2.3. The results therefore reflect a combination of pre-registered confirmatory analyses and, separately, *post hoc* exploratory analyses that describe observed patterns in the data, subject to future confirmation.

### 3.1 Behavioural analyses

Analysis of the behavioural data confirmed participants performed the behavioural tasks in line with, and in excess of, the levels to which they were trained (see section 4.2.3 here: https://osf.io/zqefx/). Accuracy rates across all no-signal (go) trials were greater than 85% (SST ​= ​98.61% ​± ​1.23%; DT ​= ​96.88% ​± ​1.91%; IT ​= ​95.10% ​± ​3.09%), as were signal trials in the DT (93.21% ​± ​3.42%) and IT (95.68% ​± ​3.26%). Successful stopping on signal trials in the SST was in accord with pre-registered target performance of ~50% (45.45% ​± ​6.03%). Reaction times (RTs) across correct trials were also within pre-registered target range for no-signal trials across all tasks (SST ​= ​456.59 ​ms ​± ​58.46 ​ms; DT ​= ​415.19 ​ms ​± ​40.00 ​ms; IT ​= ​386.25 ​ms ​± ​40.92 ​ms) and signal trials in the DT (1st response ​= ​421.55 ​ms ​± ​43.33 ​ms) and IT (396.70 ​ms ​± ​43.73 ​ms).

**Fig. 3:**
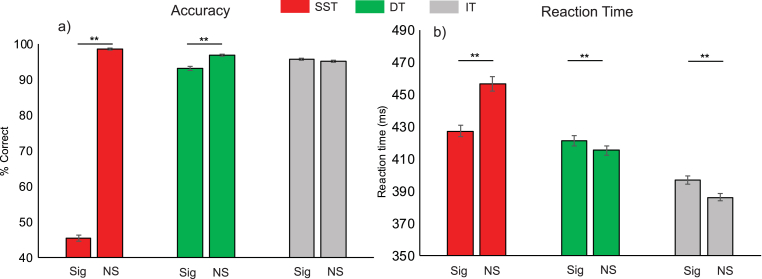
**Differences in accuracy and reaction times across tasks and trial types as revealed by repeated measures ANOVA**. Repeated measures ANOVA conducted on a) % correct and b) reaction times (RTs, in ms) across task contexts (SST ​= ​stop signal task (red), DT ​= ​double-response task (green), IT ​= ​ignore task (grey)) and trial types (signal (S) or no-signal (NS)). RTs on DT trials are for the first response. RTs on signal trials in the SST are for failed inhibition trials. Error bars ​= ​±1 within subject standard error (Loftus and Masson, 1994). ∗ ​= ​*p* ​< ​0.05; ∗∗ ​= ​*p* ​< ​0.001.

Exploratory repeated measures ANOVA revealed differences in accuracy and RTs across task contexts (SST, DT, IT) and trial types (signal, no-signal) (Fig. 3). For accuracy, significant main effects of context (F_(2,58)_ ​= ​1280.59, *p* ​< ​0.001, BF ​= ​9.46 ​× ​10^12^) and trial type (F_(1,29)_ ​= ​2394.53, *p* ​< ​0.001, BF ​= ​2.36 ​× ​10^9^) and an interaction effect (F_(1.44,41.84)_ ​= ​1788.71, *p* ​< ​0.001 (degrees of freedom Greenhouse-Geisser corrected), BF ​= ​6.32 ​× ​10^105^) were found. Of specific interest, no-signal trial performance significantly differed across task contexts (SST ​> ​DT ​> ​IT, all *p* ​< ​0.001, all BF ​> ​285.20) and within contexts, signal and no-signal trial accuracy rates differed for the SST (*p*_*0.0167*_<0.001, BF ​= ​1.35 ​× ​10^27^) and DT (*p*_*0.025*_<0.001, BF ​= ​22256.71), but not for the IT (*p*_*0.05*_ ​= ​0.108, BF ​= ​0.66). These findings potentially indicate differences in task difficulties across context. For RTs, significant main effects of context (SST, DT, IT: F_(1.43,41.36)_ ​= ​44.32, *p* ​< ​0.001, BF ​= ​5.90 ​× ​10^20^) and trial type (i.e. signal or no-signal; F_(1,29)_ ​= ​6.43, *p* ​= ​0.017, BF ​= ​0.23) and an interaction effect (F_(1.63,47.40)_ ​= ​140.20, *p* ​< ​0.001, BF ​= ​131451.61) were again observed (degrees of freedom are Greenhouse-Geisser corrected). Here, across task contexts, RTs to no-signal trials significantly differed (SST ​> ​DT ​> ​IT, all *p* ​< ​0.001, all BF ​> ​12924), consistent with the common observation of proactive or preparatory slowing in the SST (Aron, 2011; Verbruggen and Logan, 2008) and potentially higher cognitive demand in the DT in comparison to the IT. Further, within task differences revealed RTs to signal trials were longer than no-signal trials in the DT (*p*_*0.05*_ ​= ​0.006, BF ​= ​6.52) and IT (*p*_0.025_<0.001, BF ​= ​123312.11) and shorter in the SST (*p*_*0.0167*_<0.001, BF ​= ​3.20 ​× ​10^8^). Longer RTs are likely due to distraction effects (Leiva et al., 2015) and shorter RTs on stop signal trials are expected given that responses with longer RT are likely to be successfully inhibited and therefore not contribute to the RT measure (Verbruggen and Logan, 2008). Collectively, these results indicate that there are differences in control requirements beyond reactive action updating.

Additional task-specific measures were computed for use with brain-behaviour analyses and are reported in SI 2.1.3 as they are not used in analyses reported here.

### 3.2 Common and distinct cortical activity under different action updating contexts: pre-registered and exploratory analyses

Central to this investigation was to test the specificity of regions under conditions of inhibitory and non-inhibitory action updating, with a focus on the right IFG and pre-SMA. Analysis revealed right frontal dominance associated with response inhibition and left lateralisation associated with double-responding (see also SI 2.2 for additional pre-registered analyses and SI 2.3.3 for tests of laterality between pre-specified ROIs).

**Fig. 4:**
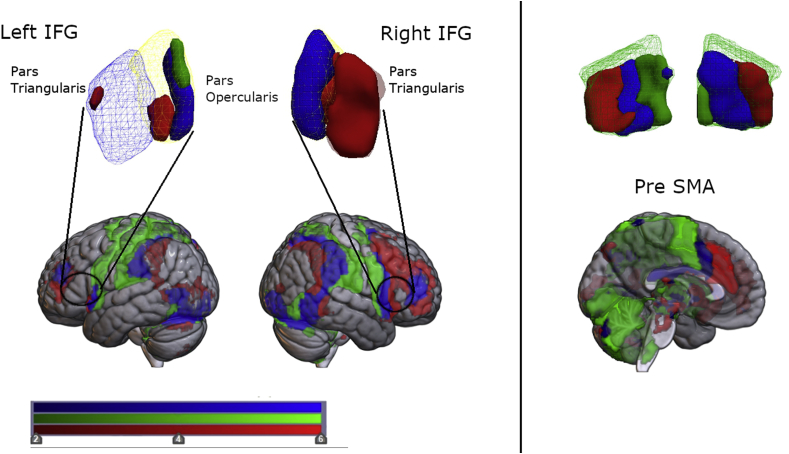
**Common and distinct regions of activity associated with different forms of action updating**. Cluster based activity significant at Z ​> ​2.3, *p* ​< ​0.05. Images are illustrated in neurological format (L ​= ​L; R ​= ​R). Red regions ​= ​activity unique to inhibitory action updating (stop signal ​> ​stop no-signal) NOT (double signal ​> ​double no-signal); green regions ​= ​activity unique to non-inhibitory action updating (double signal ​> ​double no-signal) NOT (stop signal ​> ​stop no-signal); blue regions ​= ​activity common to both types of updating (stop signal ​> ​stop no-signal) ∩ (double signal ​> ​double no-signal) for the inferior frontal gyrus (IFG) and its subdivisions (the *pars operculars* and the *pars triangularis*), and the pre-supplementary motor area (pre-SMA). Activity separated into left and right regions is presented. Scale corresponds to Z-statistic values.

Differences between inhibitory and non-inhibitory action updating were revealed with pre-registered disjunction analyses ((stop signal ​> ​stop no-signal) NOT (double signal ​> ​double no-signal) and (double signal ​> ​double no-signal) NOT (stop signal ​> ​stop no-signal)). Inhibiting a response was associated with exclusive activity in right frontal regions (Fig. 4), with 40.02% of the right IFG recruited (% of voxels within this ROI exceeding Z ​> ​2.3; see also SI Table 6). Under conditions of response inhibition, there appeared to be an anterior spread of activity in right IFG. Further exploratory analysis of the subdivisions of the rIFG revealed specialised, and more prominent, activity in the anterior right IFG, the *pars triangularis* (68.33% of this region), relative to the most posterior right IFG, the *pars opercularis* (14.74%). In comparison, no right IFG voxels were uniquely associated with non-inhibitory action updating. In the pre-SMA, disjunction analyses revealed exclusive activity under conditions of response inhibition (16.14% of this region), which appeared in the anterior portion. Conversely, more posterior activity was observed under conditions of non-inhibitory action updating (15.59%; Fig. 4b).

**Fig. 5:**
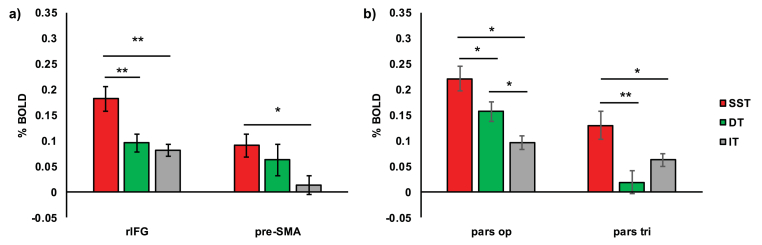
**%BOLD differences across sites and tasks as revealed by repeated measures ANOVA**. Repeated measures ANOVA conducted on %BOLD acquired from signal ​> ​no-signal contrasts for each task context for different ROIs (**a)** right inferior frontal gyrus (rIFG) and pre-supplementary motor area (pre-SMA); **b**) *pars opercularis* (pars op) *and pars triangularis* (pars tri)) and task contexts (SST ​= ​stop-signal task (red), DT ​= ​double task (green), IT ​= ​ignore task (grey)). Error bars ​= ​±1 standard error; ∗ ​= ​*p* ​< ​0.05; ∗∗ ​= ​*p* ​< ​0.001.

To further quantify this specificity a series of exploratory tests were applied to %BOLD acquired from signal ​> ​no-signal contrasts from each task (tests are considered exploratory as although contrasts and ROIs were pre-registered the interrogation of %BOLD was not). Repeated measures ANOVA between ROI (right IFG and pre-SMA) and task (SST, DT and IT) revealed significant main effects (ROI: F_(1,29)_ ​= ​19.81, *p ​<* 0.001, BF ​= ​149.32; task: F_(2,58)_ ​= ​7.42, *p* ​= ​0.001, BF ​= ​348.14), but no clear interaction effect (F_(2,58)_ ​= ​2.10, *p* ​= ​0.132, BF ​= ​0.26; Fig. 5a). Results indicate that right IFG recruitment was significantly greater than the pre-SMA (*p*_*0.05*_<0.001, BF ​= ​226.40). Further, right IFG was recruited to a significantly greater extent under SST conditions relative to DT (*p*_*0.0167*_ < 0.001, BF ​= ​38.49) and IT (*p*_*0.025*_<0.001, BF ​= ​35.44) conditions, with no difference between DT and IT requirements (*p*_*0.05*_ ​= ​0.494, BF ​= ​0.24). In the pre-SMA, %BOLD was stronger under SST conditions relative to IT (*p*_*0.0167*_ ​= ​0.020, BF ​= ​2.51) conditions, and while graded recruitment is indicated across tasks (Fig. 5a), other comparisons revealed no statistical differences in recruitment (DT vs. IT: *p*_*0.025*_ ​= ​0.123, BF ​= ​0.60; SST vs. DT: *p*_*0.05*_ ​= ​0.344, BF ​= ​0.30).

Given the differences in regional recruitment across the right IFG sub-divisions, a separate exploratory repeated measures ANOVA was conducted between ROI (*pars opercularis* and *pars triangularis*) and task (SST, DT and IT). Significant main effects (ROI: F_(1,29)_ ​= ​59.58, *p* ​< ​0.001, BF ​= ​57776.94; task: F_(2,58)_ ​= ​9.79, *p* ​< ​0.001, BF ​= ​8122.72) and a significant interaction (F_(2,58)_ ​= ​10.91, *p* ​< ​0.001, BF ​= ​2.94) were revealed (Fig. 5b). The graded activity in the *pars opercularis* (all *p* ​< ​0.012, all BFs>3.80; Fig. 5b), suggests differential recruitment depending updating requirements. Conversely, the *pars triangularis* may be especially reactive to the cancellation of actions (SST ​> ​DT: *p*_0.0167_<0.001, BF ​= ​71.72; SST ​> ​IT: *p*_0.025_ ​= ​0.030, BF ​= ​1.80), but not updating in the absence of inhibition (DT vs. IT: *p*_0.05_ ​= ​0.105, BF ​= ​0.68).

Despite differences in regional recruitment, common activity between task conditions was also evident as revealed by pre-registered conjunction analysis: (stop signal ​> ​stop no-signal) ∩ (double signal ​> ​double no-signal) (Nichols et al., 2005, Fig. 4; see also SI 2.2). Under these ‘general’ action updating conditions, shared activity was more pronounced in posterior right IFG, the *pars opercularis* (85.26% of this region), as opposed to the anterior right IFG, the *pars triangularis* (9.76%; Fig. 4, SI Table 6). Together, with the findings from the disjunction analyses, these results indicate right IFG involvement in multiple action updating demands; with the *pars opercularis* associated with general action updating, and *pars triangularis* associated with the suppression of motor responses. Common activity in the pre-SMA (27.41% of this region) is in accord with the results reported above and suggests that the pre-SMA supports both action updating and the execution of simple responses.

Collectively, these analyses show that the right IFG, and its sub-regions, are recruited to a greater extent under conditions of response inhibition (SST) relative to non-inhibitory action updating (DT) and no updating (IT) conditions. Further, the anterior spread of activity associated with response inhibition suggests the *pars triangularis* may be particularly important for response inhibition, relative to the execution of actions. However, right IFG specialisation is far from complete, since less pronounced %BOLD in DT and IT, relative to SST, does not imply no involvement (de Hollander et al., 2014; Van Horn and Poldrack, 2009).

### 3.3 Exploratory analyses of subcortical pathways involved in response execution and response inhibition

The three putative action-control pathways (direct, indirect and hyperdirect) involve a specific pattern of activity according to excitation/inhibition relationships between the structures involved (Fig. 2; Albin et al., 1989; Alexander and Crutcher, 1990; DeLong, 1990; Nambu et al., 2002). The following analyses explore the correspondence between this theoretical pattern of activity and that observed. In particular, we explored the spatial distribution and interrelationships between ROIs under relevant behavioural conditions and their consistency with the action control pathways. Our pre-registered protocol described assessment of activity within structures comprising the pathways and hypothesised that this would conform to context dependent patterns. However, the implementation of these analyses were not pre-registered and should be considered exploratory.

#### 3.3.1 Spatial distribution of activity and interrelations between ROIs

##### 3.3.1.1 Regions identified

A two-way ANOVA and t-tests were used to assess the spatial distribution of activity across cortical (preSMA and IFG) and subcortical (BG and THAL) ROIs under behaviourally relevant conditions. This involved factors of condition (execution vs. inhibition) and ROI (16 levels; data drawn from bilateral masks of IFG, pre-SMA, STR, GPe, GPi, STN, SN, THAL). A significant interaction effect (condition ​× ​site F_(4.77,138.53)_ ​= ​15.80, *p* ​< ​0.001, BF ​= ​3.86 ​× ​10^44^) indicated differential recruitment of ROIs when responses were executed or inhibited (degrees of freedom are Greenhouse-Geisser corrected).

**Fig. 6:**
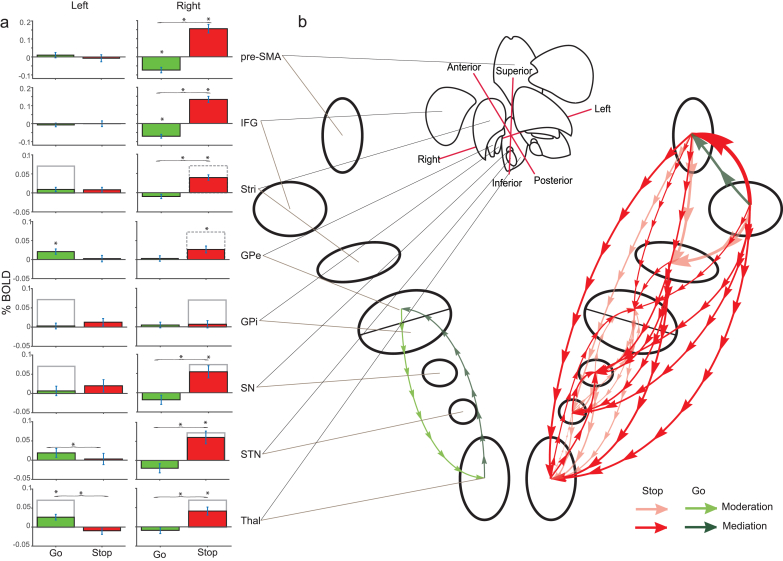
**Functional activations and interrelations between cortical and subcortical regions under conditions of response execution and response inhibition as revealed by moderator/mediator partial analyses.** A) Bar charts indicating %BOLD drawn from each ROI under either response execution (go; green) or response inhibition (stop; red). Error bars are ± 1 standard error. These represent analyses summarised in Table 1. ∗ indicates significant difference from baseline and where accompanied by parentheses indicates significant difference between stop and go conditions. Expected patterns of activity based on the pathways model are indicated by the grey bar outlines: solid outlines indicate regions considered part of either direct (left) or indirect (right) pathways, whereas dashed outlines indicate the hyper-direct pathway. **B)** Partial (moderator/mediator) analyses. The direction of the relationship is shown by the arrows and the strength of the relationship demonstrated by the width of the arrow (log 10 of the change in BF). Green arrows refer to response execution and red arrows refer to response inhibition conditions. The upper cartoon provides an anatomical visualisation of the relative position of structures based on atlases.

Following up on this interaction, activity appeared strongly lateralised (see Table 1 and Fig. 6). Responses executed with the right hand were associated with left-lateralised subcortical dominance, specifically in the THAL and GPe. Conversely, in cortical sites, response execution was associated with a relative right-lateralised suppression -; a pattern of activity opposite to the upregulation commonly identified in these regions when responses are inhibited (Table 1). While this suppression could be reflective of neuronal inhibition, it is also possible that this is due to the magnitude of activity associated with response inhibition events, against which those associated with response execution are contrasted (e.g. double signal ​> ​stop signal), within the compound contrast set. Activity associated with response inhibition was strongly right-lateralised in both cortical and subcortical regions. Generally, across ROIs, activity was increased when responses were to be inhibited vs. executed, particularly in the right hemisphere (Table 1).

**Table 1:** Simple effects analyses of %BOLD for left and right cortical and subcortical regions under conditions of response execution and response inhibition.

ROI		Execution vs. inhibition				Execution				Inhibition			
		t	df	*p*	BF	t	df	*p*	BF	t	df	*p*	BF
Left	pre-SMA	0.52	29	0.61	0.22	0.68	29	0.50	0.24	−0.37	29	0.71	0.21
	IFG	−0.26	29	0.79	0.20	−0.73	29	0.47	0.25	−0.04	29	0.97	0.19
	STR	0.08	29	0.93	0.20	1.66	29	0.11	0.66	1.27	29	0.22	0.40
	GPe	1.22	29	0.23	0.38	**3.01**	**29**	**0.01**	**7.68**	0.31	29	0.76	0.20
	GPi	−0.60	29	0.56	0.23	0.39	29	0.70	0.21	1.34	29	0.19	0.43
	SN	−0.49	29	0.63	0.22	0.54	29	0.59	0.22	1.26	29	0.22	0.40
	STN	0.61	29	0.54	0.23	1.63	29	0.11	0.63	0.21	29	0.84	0.20
	THAL	**2.16**	**29**	**0.04**	**1.46**	**3.46**	**29**	**0.002**	**20.87**	−1.01	29	0.32	0.31
Right	pre-SMA	**−6.08**	**29**	**<0.001**	**14039.59**	**−4.84**	**29**	**<0.001**	**599.72**	**6.68**	**29**	**<0.001**	**62921.33**
	IFG	**−7.64**	**29**	**<0.001**	**668686.28**	**−6.81**	**29**	**<0.001**	**87265.65**	**7.96**	**29**	**<0.001**	**1454887.67**
	STR	**−4.12**	**29**	**<0.001**	**99.94**	−1.86	29	0.07	0.89	**5.52**	**29**	**<0.001**	**3369.02**
	GPe	−1.51	29	0.14	0.54	0.39	29	0.70	0.21	**2.98**	**29**	**0.006**	**7.12**
	GPi	−0.12	29	0.91	0.20	0.64	29	0.53	0.23	0.70	29	0.49	0.24
	SN	**−2.69**	**29**	**0.01**	**3.93**	−1.55	29	0.13	0.56	**3.46**	**29**	**0.002**	**21.07**
	STN	**−2.86**	**29**	**0.008**	**5.53**	−1.72	29	0.10	0.72	**3.64**	**29**	**<0.001**	**31.30**
	THAL	**−2.79**	**29**	**0.01**	**4.84**	−1.11	29	0.28	0.34	**3.99**	**29**	**<0.001**	**72.37**

Results are presented for each region of interest (ROI) for both the left and right hemispheres, separately. t ​= ​t-statistic, df ​= ​degrees of freedom, *p* ​= ​*p*-value, BF = Bayes Factor. Results with significant *p*-values and associated Bayes Factors are presented in bold.

##### 3.3.1.2 Relationships between regions identified

The preceding analyses demonstrate patterns of context specific activity but are silent to the interrelationships between structures. The simplest way to test for these correlational relationships was to apply a series of moderator and mediator analyses (Baron and Kenny, 1986; Judd et al., 2001), appended by Bayesian equivalents (Table 2). While these analyses do pertain to directions of influence, all causal inferences and outcomes should be qualified as based on exploratory correlational evidence. ROIs which demonstrated significant %BOLD (*p* < 0.05 or BF > 3) under conditions of response execution or response inhibition (Table 1; conditions analysed separately) were entered as covariates for analysis of other ROIs which also demonstrated significant %BOLD (*p* < 0.05 or BF>3; Table 2). A covariate ROI can be said to exert a mediating influence over a target region if the original difference is eliminated (i.e. *p* > 0.05, or BF < 1/3), but exerts a moderating influence if the original difference is reduced but its significant status remains (i.e. *p* < 0.05 or BF > 3). It should be noted, however, that while a decision criterion (e.g. p < 0.05) is required for interpretation, this criterion is to some extent arbitrary.

**Table 2:**
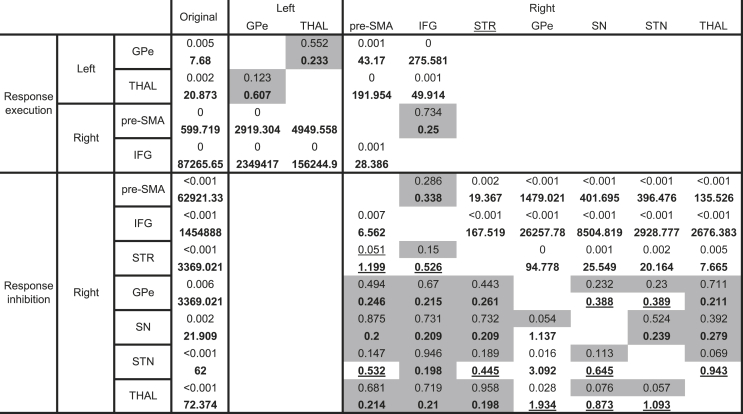
Moderator and mediator analyses under conditions of response execution and response inhibition.

Summary of the *p*-values and Bayes Factors (bold) from the simple effects analysis of condition and region of interest and how they are influenced by the addition of covariates. The original values correspond to those yielded from the simple effects analysis (where *p*<0.05 and BF>3), as presented in Table 1. The table can be read from left to right, where the regions of interest (ROI) in each column are the covariate added to the ROI in each row. Resulting values in grey represent instances where mediation has occurred, where the addition of the covariate has reduced the BF to <1/3 and *p* to >0.05. Values underlined represent moderation where the addition of the covariate has reduced the BF to <3 but >1/3 and *p*-values can maintain their significant status and BF remains >1/3.

When responses were executed, four regions demonstrated significant differences from baseline (right pre-SMA, right IFG, left GPe, left THAL; Table 1). The interrelations between ROIs appeared divided between cortical and subcortical regions. Subcortically, the left THAL and left GPe expressed a mutual mediating interrelationship, where addition of either as a covariate explained the activation of the other (Table 2). Cortically, right IFG was found to mediate right pre-SMA, but the pre-SMA was found to moderate activity in the right IFG. The same directional influence from right IFG to pre-SMA was also identified when responses were to be inhibited (Table 2). However, the similarity in patterns of activity across behavioural conditions suggests that these relationships may be independent of control requirements (but see Zhang and Iwaki, 2019, who identified causal activity from IFG to SMA, modulated by inhibition).

Under conditions of response inhibition, mutual interdependency was demonstrated between right cortical and subcortical structures (Table 2, Fig. 6). The right IFG and right pre-SMA exerted complete mediation over observed BG and THAL activity. However, subcortical activity moderated IFG and pre-SMA activity, consistent with a cortical to subcortical drive. The general pattern of subcortical activity also indicated a directional relationship that fits with expected motor physiology during top-down control; mediating effects were generally found downstream according to their proposed directions of influence under the pathway models (from STR to THAL; Albin et al., 1989; Alexander and Crutcher, 1990; DeLong, 1990; Nambu et al., 2002), while moderating effects were generally found upstream (from THAL to STR). The GPe appeared to be an exception to this pattern, where all other subcortical regions mediate GPe activity, the GPe itself only exerts moderating influence on other structures. Speculatively, it is possible, that right GPe is not essential for implementing inhibition *per se*, but might be important for the integration or communication of signals between regions when action plans are updated (Suryanarayana et al., 2019). This proposition could also explain the apparent importance of the left GPe in response execution even though it is not classically considered important for the direct pathway (see Table 1). Also of note, the right THAL, which, when responses are to be inhibited, appears to exert a mediating influence on all other structures (with the exception of the GPe). Again, speculatively, the strength and influence of these activations might be evidence of potential feedback mechanisms important or ensuring responses are inhibited after initial suppression.

#### 3.3.2 Consistency between observed activity and hypothesised pathways

The following analyses aimed to assess the consistency between the pattern of observed activity and that expected according to the pathways. Models representative of each pathway were applied to the compound contrast data. To confirm that any correspondence was not the result of over-fitting, data were also applied to the theoretically inappropriate models.

**Fig. 7:**
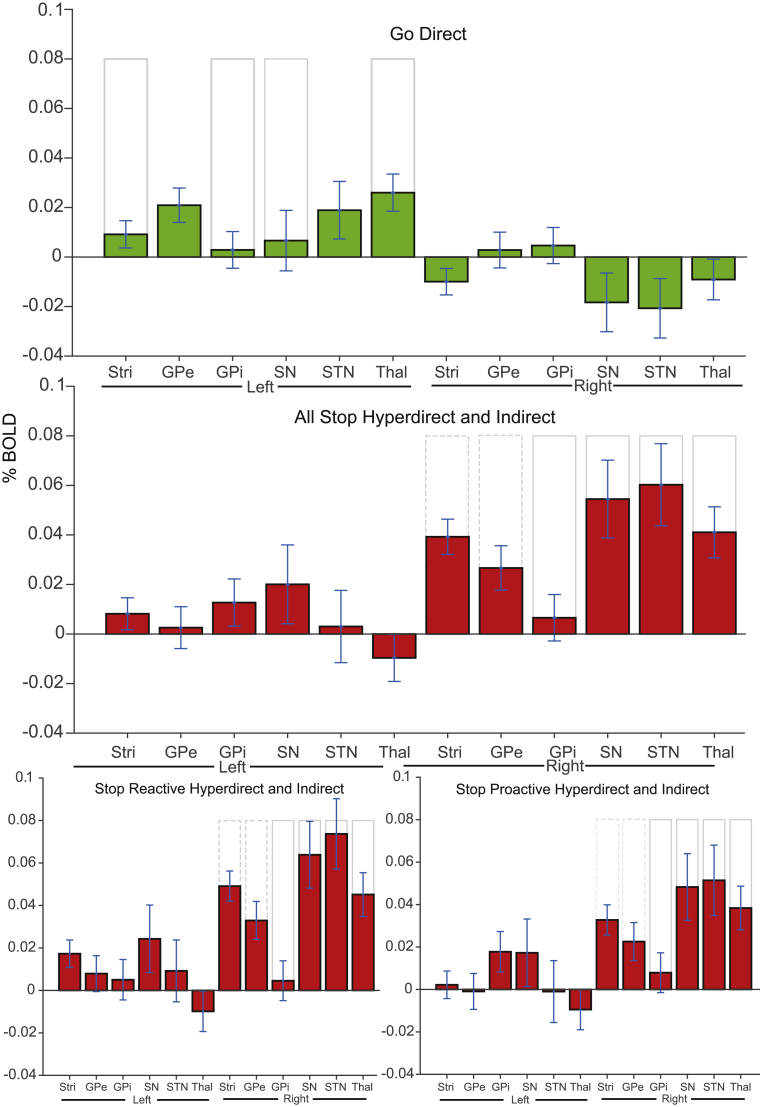
**%BOLD from ROIs constitutive of the putative pathways**. %BOLD extracted from bilateral ROIs contributing to the direct, indirect and hyperdirect pathways. Error bars ±1 standard error. The grey outlines indicate the theoretical (expected) pattern of the data under each model for regions hypothesised to be involved in each pathway (height is arbitrary). Dashed lines indicate regions theoretically involved in both the indirect and hyperdirect pathways.

Results shown in Table 3 and Fig. 6, Fig. 7 broadly support the proposed models, their laterality and their functional specificity with high levels of consistency between expectations of where activity should occur and that observed under behaviourally relevant conditions. Fits were inconsistent when the behaviourally opposing contrasts (i.e. those theoretically incorrect) were applied. Strong correspondence was evident when response execution data were applied to the direct model and when response inhibition data were applied to both the indirect and hyperdirect models. However, the division of inhibitory contrasts into proactive and reactive (i.e. contrasts reflective of preparatory inhibition and the active stopping of responses, respectively) did not show differentiation between the indirect and hyperdirect pathways. Given that the indirect pathway is thought to support the tonic suppression of actions (Aron, 2011; Majid et al., 2013; Zandbelt et al., 2013), we expected greater consistency with proactive data than reactive data. However, the opposite was found. As illustrated in Fig. 7, under reactive, relative to proactive, conditions, activity in most ROIs (with the exception of the GPi) showed greater consistency with the indirect pathway. This may be due to reactive contrasts resulting in generally larger %BOLD changes than proactive contrasts. In support of this, a pre-registered analysis revealed stronger %BOLD within the right IFG under reactive vs. proactive inhibitory control (t_(29)_ ​= ​3.22, *p* ​= ​0.003, BF ​= ​12.10). These results are likely due to the active nature of reactive stopping which is likely more hemodynamically demanding than implementing slower proactive control. Additionally the lack of differentiation can also be attributed to there only being two structures (GPe and SN) which are involved in the indirect and not hyperdirect pathways, whereas all other structures are expected to respond in the same way for both pathways (Nambu et al., 2002). These considerations do, however, limit the extent to which the reactive-hyperdirect vs. proactive-indirect correspondence question can be posed using fMRI and within this data.

**Table 3:** Correspondence between pattern of activations in subcortical ROIs and the pathways models.

		Pathway		
		Direct	Indirect	Hyperdirect
Contrast condition	Execution	**21.945**	0.002	0.056
	Inhibition All	1.917 ​× ​10^−9^	**3.216** ​× ​**10**^**11**^	**558.224**
	Inhibition Pro	4.666 ​× ​10^−6^	**1.482** ​× ​**10**^**8**^	**2681.154**
	Inhibition Reac	1.345 ​× ​10^−10^	**1.539** ​× ​**10**^**12**^	**91.459**

Bayes Factors for theoretically appropriate assignment of the data applied to each model (bold values; e.g. response execution contrasts applied to the direct pathway model) and theoretically inappropriate models were those to which data assignment did not match theory (e.g. response inhibition and direct pathway model). Bayesian t-tests were used to assess the consistency between %BOLD across subcortical ROIs for each pathway and the models used to represent each pathway. Results are presented for response inhibition are computed across all inhibitory contrasts (Inhibition All) and subdivided into proactive (Pro) and reactive (Reac) contrasts.

Also inconsistent with expectations was activity in the left GPe during response execution; a region not classically considered important for the direct pathway (Albin et al., 1989; Alexander and Crutcher, 1990; DeLong, 1990; Nambu et al., 2002, Fig. 2). Speculatively, this activation may result from involvement in action sequencing (Chan et al., 2005; see also Nambu, 2008) as required for double-responding, or could result from imprecision in the localisation of activity in such a small region (possibly compounded by the use of the pre-registered spatial smoothing kernel; de Hollander et al., 2015). An absence of right GPi activation under conditions of response inhibition was also unexpected as the GPi is part of both the hyperdirect and indirect pathways. Again speculatively, this could be due a lack of vascular innovation or responsiveness (e.g. Lai et al., 2014).

To summarise, our results indicate cortical and subcortical lateralisation of action control. The pattern of activity revealed largely corroborate the pathways models. While left-hemisphere dominance was exhibited under conditions of response execution with the right hand, these analyses suggest right-hemisphere dominance that extends subcortically under conditions of response inhibition.

## 4 Discussion

This study aimed to establish the neuroanatomical distribution of inhibitory and non-inhibitory action updating and explored the subcortical pathways hypothesised to underlie response inhibition and response execution. Our results suggest that a broad network supports general processes common to different forms of action updating, with more specialised (and lateralised) sub-units of activity supporting inhibitory control.

The use of a context-cueing paradigm (Fig. 1) in combination with fMRI allowed us to establish regional activation associated with inhibitory action updating (using a SST) and non-inhibitory action updating (using a DT). However, while the DT and SST were matched as closely as possible in terms of stimuli and the requirement to update action plans, differing only in cognitive inhibition, it is possible that inhibition is more effortful and has a different time course in the SST compared with the DT. Therefore, it is always possible to attribute differences in observed activity to such differences in effort or dynamics. Even so, in accordance with previous work (Chatham et al., 2012; Dodds et al., 2011; Erika-Florence et al., 2014; Hampshire, 2015; Tabu et al., 2011), the requirement to update action plans (in both the SST and DT) was associated with common activity across both cortical and subcortical regions, including the pre-SMA and posterior right IFG, the *pars opercularis* (see Fig. 5). Distinct forms of action updating (SST or DT) were associated with differential patterns of unique activity. Within the IFG, inhibiting a response revealed exclusive frontal lobe activation, specifically in the anterior right IFG, the *pars triangularis* (Fig. 5b). This observation is inconsistent with suggestions that a specialised inhibitory module lies within the posterior right IFG, the *pars opercularis* (Aron et al., 2014a, Aron et al., 2014), but is consistent with evidence showing structural changes in *pars triangularis* associated with inhibitory control training (Chavan et al., 2015). Here, further investigation is required which may benefit from recent developments in MRI technologies that allow for more accurate and finer resolution analyses of activation topography (e.g. multiband sequences, higher number of head coil channels, improved motion correction).

Previous studies have also indicated no functional specialisation in right IFG associated with response inhibition (e.g. Chatham et al., 2012; Dodds et al., 2011; Erika-Florence et al., 2014; Hampshire, 2015; Hampshire et al., 2010; Tabu et al., 2011). The *pars triangularis* specificity identified here could well be due to task differences, with the functional disparity explained by the possible hierarchical organisation of the frontal lobes along the caudal-rostral axis; with more caudal regions supporting concrete information about actions, and the more rostral, supporting more abstract action goals (Badre and D’Esposito, 2009; Botvinick, 2008). As such, the more caudal, *pars opercularis*, could be important for action updating requirements more generally (SST and DT), with the more rostral, *pars triangularis*, receptive to ambiguous responding (Levy and Wagner, 2011) as per SST instructions (i.e. to go, but stop where possible) or cancelling ongoing actions, which may be greater when embedded in a context-cueing paradigm.

While the right IFG appears to be more specialised for inhibitory control in comparison to the pre-SMA (Fig. 4a), both cortical regions appeared to be important for its implementation at a subcortical level. Right IFG and right pre-SMA were found, in exploratory analyses, to mediate all downstream BG and THAL activity when responses were stopped. Our evidence suggests that right IFG exerts mediating influence over pre-SMA, suggestive of overall control under conditions of response inhibition (see also Duann et al., 2009; Rae et al., 2015). However, the moderating influence of right IFG by pre-SMA is also indicative of a mutually interdependent relationship; indeed, evidence indicates parallel activation of these regions when actions are cancelled (Allen et al., 2018). The contrasts indicated greater activity in the right *pars triangularis* for response inhibition, relative to other updating conditions, with less activity in right *pars opercularis* and the left IFG. There was, however, additional activity for these contrasts outside the pre-registered regions targeted by the design (as seen in Fig. 4). As an exploratory observation, beyond pre-registered ROIs the pattern of inhibition-specific activation extended anteriorly and could be interpreted as consistent with a frontal inhibitory control network bordered by the inferior frontal sulcus and pre-SMA.

Subcortically, exploration of BG and THAL revealed patterns of activity consistent with, and suggestive of, a potential functional mechanism in which execution of right-handed responses is implemented by a left-hemispheric network, which is actively blocked by a right-lateralised inhibitory network (Fig. 6). Indeed, right-lateralised BG and THAL demonstrated significant increase in %BOLD when responses were stopped, and were reliably stronger when responses were inhibited vs. executed (with the exception of the GPe and GPi; Table 1). The pattern of activity identified fits with motor physiology and is consistent with the pathways models; mediating effects were generally found downstream, from STR to THAL. However, the pathway models likely oversimplify the interplay between regions; apparent categorisation of relationships as moderating and mediating to some extent can be dependent on the magnitude of effects observed relative to decision criteria (e.g. *p* ​< ​0.05) and independently of covariance relationships, and the temporal dynamics of interrelations between structures were not taken into account in these analyses. Even so, our data indicates interdependencies, such as upstream relationships (from THAL to STR), not described by the classic models, and suggest areas for future investigation. Similarly, mutual interdependent effects were also found under conditions of response execution between left GPe and left THAL. Such mutual interaction between regions hints at the presence of continual feedback loops (Chan et al., 2005; Graybiel, 2005; Smith et al., 2004) that might operate to ensure response preparation and/or continuous movement.

BG and THAL constitutive of each pathway generally demonstrate significant change in %BOLD under the relevant response control conditions. However, the region that was most difficult to reconcile with its theoretical role in the putative pathways was the GPe. This structure is not classically considered part of the direct pathway, yet here, left GPe was found to be significantly recruited under conditions of response execution, along with the left THAL, with a mutually dependent relationship between them. Under conditions of response inhibition, the right GPe was found to be correlationally under the governance of all BG and THAL regions, but exerted minimal influence itself, even though this structure is hypothesised to be important to the indirect pathway (Fig. 2).

The GPe has been considered a relay hub given its widespread interconnectivity with other BG nuclei (Suryanarayana et al., 2019); potentially important for the integration or communication of signals between regions when action plans are updated. Specifically, it has been suggested that the GPe may play a role in the execution of response sequences (Chan et al., 2005; see also Nambu, 2008), which is required on signal trials in both the SST and DT. Recent work has highlighted the GPe as crucial to action selection, hypothesising that activity from the STN to SN (components of the hyperdirect pathway) might initiate a ‘pause’ so that selective cancellation of actions can occur via the GPe to STR (Mallet et al., 2016; Suryanarayana et al., 2019). Indeed, STN has been found to be the main excitatory input to the GPe (Hegeman et al., 2016). Further, direct projections from the cortex (Chen et al., 2015; Milardi et al., 2015; Saunders et al., 2015) to the GPe have been identified, but the importance of this has not yet been established.

The laterality in response control identified here extends previous proposals of motor laterality in the human brain. An interesting direction for future research would be to explore the potential that this lateralisation is associated with hand used to execute responses and handedness, as all participants in the current study were right-handed and used their right hand to perform the task. Previous research has identified contralateral activation of BG during hand movements (Solodkin et al., 2001), and although the causal mechanisms between lateralisation of cortical function and handedness are unknown, it is possible they are supported by common mechanisms. This could be readily tested using a mixed-model design with right and left-handers performing bilateral versions of tasks similar to those employed here, potentially confirming these exploratory findings.

Functional MRI as a technique has been criticised for its inability to detect small changes in BOLD responses (Manova et al., 2009), yet our approach enabled us to delineate the BG and THAL activity and suggest how the pathways might be revised. It is likely that this may be due to the incorporation of the novel compound contrast analyses which is theoretically more robust than the common practice of choosing individual representative contrasts. Such methods could potentially be further developed to provide functional biomarkers of BG disorders and help aid targets for therapies. More generally, the approach of computing all possible contrasts of interest and taking the mean, utilises the advantages of averaging by eliminating factors that are not of interest (multiple baselines) and focussing the analysis on common factors of interest. This can be applied to improve sensitivity beyond the domain of cognitive control.

In conclusion, the evidence discussed here suggests that a widely distributed fronto-parietal network of activity underlies general action updating processes (Hampshire, 2015; Hampshire and Sharp, 2015) which also contains regions specific for response inhibition. Inhibition of responses also engages a right lateralised network that extends to subcortical structures, which exploratory evidence indicates may block action-related activity.

## Data and code availability statement

All data and code used in the study, for which we have the legal rights to disseminate, are openly available in the public domain at https://osf.io/zbk3p/.

## Ethics statement

Written informed consent was obtained from all participants, and all procedures were approved by the research ethics committee at the School of Psychology, Cardiff University.

## Funding

This study was supported by 10.13039/501100000268Biotechnology and Biological Sciences Research Council grant code BB/K008277/1, 10.13039/100010269Wellcome Trust grant code 104943/Z/15/Z and 10.13039/501100000781European Research Council Consolidator Grant 647893 (C.D.C.).

## CRediT authorship contribution statement

**Leah Maizey:** Conceptualization, Methodology, Software, Validation, Formal analysis, Investigation, Resources, Data curation, Writing - original draft, Writing - review & editing, Visualization, Project administration, Funding acquisition. **C. John Evans:** Conceptualization, Methodology, Formal analysis, Investigation, Resources, Data curation, Writing - review & editing, Supervision. **Nils Muhlert:** Conceptualization, Methodology, Software, Validation, Resources, Writing - review & editing, Visualization. **Frederick Verbruggen:** Conceptualization, Methodology, Software, Resources, Writing - review & editing, Supervision, Funding acquisition. **Christopher D. Chambers:** Conceptualization, Methodology, Validation, Formal analysis, Resources, Writing - review & editing, Supervision, Project administration, Funding acquisition. **Christopher P.G. Allen:** Conceptualization, Methodology, Software, Validation, Formal analysis, Investigation, Resources, Data curation, Writing - original draft, Writing - review & editing, Visualization, Project administration, Funding acquisition.

## Declaration of competing interest

The authors have no competing interests.

## Footnotes

1 For 2 participants, fieldmaps were not used due to poor quality. Although, as pre-registered, fieldmaps were acquired in a later session, these were omitted from the analysis since the information required for un-warping is dependent on participant head geometry and orientation with the scanner at the time of the scan.
