# Supplementary material for: Cortical and subcortical functional specificity associated with response inhibition
Source: Neuroimage. 2020 Oct 15;220:117110. doi: 10.1016/j.neuroimage.2020.117110 (PMC7573537; doi:10.1016/j.neuroimage.2020.117110)
Supplement: Multimedia component 1 [file mmc1.docx]

Cortical and subcortical functional specificity associated with response inhibition: supplementary information

Leah Maizey, C. John Evans, Nils Muhlert, Frederick Verbruggen, Christopher D. Chambers & Christopher Allen.

Supplementary materials are ordered as per the main manuscript. The corresponding pre-registration document and amendments can be found here:

<https://osf.io/zqefx/> and <https://osf.io/27gmh/>. The first section of the supplementary information (SI) contains methodological detail not included in the main manuscript. The second section reports the results for pre-registered analyses beyond those described in the main text (SI section 2): i) behavioural analyses (SI section 2.1), ii) common and distinct cortical processes under different action updating conditions (SI section 2.2), and iii) region of interest (ROI) analyses aimed at exploring the relationships between blood oxygen level dependent (BOLD) signal and task performance in the stop-signal task (SST; SI section 2.3.1) and the double-response task (DT; SI section 2.3.2). Alternative analytical decisions and results related to the exploratory analysis of subcortical regions and action control pathways described in the manuscript are reported here: <https://osf.io/zkq7h/>

**Table of Contents**

[**1.**](#_heading=h.30j0zll) **Methods 3**

[1.1.](#_heading=h.1fob9te) Excluded participants 3

[1.2.](#_heading=h.3znysh7) Technical detail 3

[1.3.](#_heading=h.2et92p0) Physiological measures 4

[1.4.](#_heading=h.tyjcwt) Procedure 4

[1.5.](#_heading=h.3dy6vkm) Regions of interest 5

[1.6.](#_heading=h.1t3h5sf) Compound contrasts 6

1.7. Temporal signal to noise ratio 9

[**2.**](#_heading=h.4d34og8) **Analysis and results 9**

[2.1.](#_heading=h.2s8eyo1) Behavioural results summary 9

[2.1.1.](#_heading=h.17dp8vu) Accuracy 10

[2.1.2.](#_heading=h.3rdcrjn) Reaction times 11

[2.1.3.](#_heading=h.26in1rg) Task specific measures 12

[2.1.3.1.](#_heading=h.lnxbz9) Stop-signal task 12

[2.1.3.2.](#_heading=h.35nkun2) Double response task 13

[2.2.](#_heading=h.1ksv4uv) Common and distinct cortical processes under different action-updating contexts: pre-registered and exploratory analyses 14

[2.3.](#_heading=h.44sinio) Brain-behaviour relationships 19

[2.3.1.](#_heading=h.2jxsxqh) Stop signal task 19

[2.3.1.1.](#_heading=h.z337ya) Correlation between %BOLD and SSRT 20

[2.3.1.2.](#_heading=h.3j2qqm3) Relationship between stop signal delay and %BOLD 23

[2.3.1.3.](#_heading=h.1y810tw) Difference between %BOLD on successful vs. unsuccessful stop trials 26

[2.3.2.](#_heading=h.4i7ojhp) Double-response task analyses 27

[2.3.2.1.](#_heading=h.2xcytpi) Correlation between %BOLD and DRT2 27

[2.3.2.2.](#_heading=h.1ci93xb) Correlation between %BOLD and the size of the PRP 28

[2.3.2.3.](#_heading=h.3whwml4) Relationship between stimulus onset asynchrony and %BOLD 29

[2.3.3.](#_heading=h.2bn6wsx) Lateralisation analyses 32

2.3.3.1. Lateralisation of ROIs 32

2.3.3.2. Lateralisation of cortical clusters 33

2.4. Temporal Signal to noise maps 34

2.5. Effect size estimates for interrelations between ROIs analyses 36

[**3.**](#_heading=h.3as4poj) **References 37**

# **Methods**

For inclusion and exclusion criteria, task instructions and details of training, see pre-registration document and amendments (<https://osf.io/zqefx/> and <https://osf.io/27gmh/>).

## Excluded participants

In total, 8 participants were excluded (38 participants tested, final sample N = 30). 1 participant was excluded for failing to meet the performance benchmarks set in training^[[1]](#footnote-1)^, 1 participant failed safety screening prior to the scan session, 3 participants were excluded for failing to meet the performance benchmarks set in the scan session^[[2]](#footnote-2)^, 1 participant was excluded for excess motion in the scanner, 2 participants voluntarily withdrew from the study.

Single run fMRI and corresponding behavioural data were excluded for 4 participants due to excess head motion (more than 1 voxel in any direction) as pre-registered (see section 9 here: <https://osf.io/zqefx/> and clarification point 3 here: <https://osf.io/27gmh/>). This data was also excluded from behavioural analyses and brain-behaviour analyses (SI sections 2.1 and 2.3, respectively).

## Technical detail

The context-cueing paradigm was programmed in Psychophysics toolbox in MATLAB ([www.psychtoolbox.org](http://www.psychtoolbox.org); Mathworks, Natick, MA; Brainard, 1997). All stimuli were presented in the centre of a screen at the rear of the bore using a Canon Xeed SX60 projector system (1024x768 pixel, refresh rate = 60Hz, Canon, UK). The presentation screen was located ~47cm away from the participants’ eyes and viewed through a mirror mounted onto the MR-head coil (located ~12cm away from the participants’ eyes). Visual angle = 1.75˚ × 3.69˚ (arrow stimuli) and 1.75˚ × 1.75˚ (fixations). Responses were collected using Lumitouch^TM^ MRI compatible response boxes (Photon Control Inc, Canada).

## Physiological measures

Measures relating to physiological monitoring were acquired throughout the scan session as a means to remove associated artefacts commonly found in mid-brain regions and to improve the signal to noise ratio of the acquired functional data (Bright and Murphy, 2013; Brooks et al., 2013). All physiological data were continuously acquired during each scan session using a nasal cannula, pulse oximeter and respiration bellows. The nasal cannula was connected to CO2 and O2 gas analysers (AEI Technologies, PA, USA). Measures were sampled at 500Hz and logged via a Power1401 (CED, Cambridge, UK) using Spike 2.7 (CED, Cambridge, UK). Physiological regressors (cardiac rate, respiration rate, O2 troughs and end-tidal CO2) were removed from Echo-Planar Imaging (EPI) data prior to pre-processing (Bright and Murphy, 2013).

## Procedure

Participants were offered a short comfort break after 4 fMRI runs. An additional fieldmap (prescription as per main manuscript) was acquired prior to this break. As per our pre-registration documents if susceptibility weighted images (SWIs) were not acquired (e.g. due to technical issues), or inadequate for use (e.g. due to motion artefacts), they were re-acquired at a later date. In total, SWI scans were re-acquired for 15 participants. Where 2 sets of SWIs were acquired, those in which the subthalamic nucleus (STN) was most readily identifiable was used to create this ROI (SI section 1.5.).

Deviations from pre-registered document:

Slice time correction was not pre-registered as pre-processing step for fMRI data. However, this has now been used to re-analyse all fMRI data after reviewer requests.

Further, we pre-registered that poor quality / inadequate fieldmaps would be re-acquired at a later date. This was done for 2 participants, but we since decided not to use fieldmap correction for these individuals since the information required for un-warping is dependent on participant head geometry and orientation with the scanner at the time of the scan.

## Regions of interest

Deviations from pre-registered document: In our pre-registered document (<https://osf.io/zqefx/>) we stated that all basal ganglia ROIs would be identified using the Harvard-Oxford Subcortical atlas (Desikan et al., 2006; Frazier et al., 2005; Goldstein et al., 2007; Makris et al., 2006), with the exception of the STN which would be localised manually using each participant’s SWI. However, prior to data analysis the decision was taken to localise all subcortical structures (with the exception of the Thalamus (THAL)) with the Atlas of the bAsal basal ganglia (ATAG; Keuken et al., 2014; all ROIs thresholded at 25%). The reasons for this change were: 1) the Harvard-Oxford Subcortical Atlas does not differentiate between the globus pallidus externa (GPe) and globus pallidus interna (GPi), which was necessary to explore activity with respect to the pathways models; 2) the ATAG contains an ROI of the STN, whereas the Harvard-Oxford Atlas does not; and 3) The ATAG was published after our pre-registration document. Analyses reported in the manuscript still make use of the manually identified STN for each participant using SWIs, but analyses using STN identified using the ATAG are reported throughout the SI. These analyses are reported in addition to those manually identified STN and are included in our pre-registered brain-behaviour analyses (SI section 2.3) and exploratory analyses of the putative action control pathways (see: [<https://osf.io/zkq7h/>)](https://osf.io/tbk74/)).

Further detail pertaining to identification of STN:

Number of voxels in atlas defined STN: left = 6 voxels; right = 8 voxels.

Number of voxels in manually identified STN: left = 9.2 ± 3.54 voxels; right = 11.00 ± 4.81 voxels.

The % of overlapping voxels between atlas based and manually derived STN: left STN = 35.00% ± 32.27%; right STN = 38.33% ± 24.99%.

Further detail pertaining to identification of substantia nigra (SN):

Although this region was identified using the ATAG atlas, voxels from the SN were removed when the STN was manually defined.

Number of voxels in atlas defined SN: left = 49 voxels; right = 49 voxels.

Number of voxels in SN when voxels identified as STN removed: left = 47.27 ± 2.32 voxels; right = 47.47 ± 2.70 voxels.

The % of overlapping voxels between atlas defined SN and that with voxels identified as STN excluded: left SN = 96.46% ± 4.73%; right SN = 96.87% ± 5.51%.

## Compound contrasts

In the manuscript we reported a compound contrast approach that made use of all (reasonable) contrasts. In total, 199 contrast combinations were computed and categorised as reflective of the direct pathway, indirect pathway, hyperdirect pathway, or unclear. Each contrast was independently categorised by 3 researchers (LM, NM and CA; see SI table 1). If categorisation was not agreed by the 3 researchers, the contrast was labelled as unclear. Of these 199 contrasts, 131 were clearly categorised and were run in separated GLMs as reported in the manuscript (*separate* GLM approach). In another exploratory approach we ran all 199 contrasts in a single GLM (*single* GLM approach; see <https://osf.io/zkq7h/> for further details). Unclear contrasts were included here as all events were specified in the single GLM approach. Subsequent analysis of patterns of %BOLD excluded unclear contrasts as these were not readily categorised as reflective of either response execution (direct pathway) or response inhibition (indirect or hyperdirect pathways).

| Pathway categ. | Contrast |  |  |  |  | Pathway categ. | Contrast |  |  |  |
| --- | --- | --- | --- | --- | --- | --- | --- | --- | --- | --- |
| Response execution (direct pathway) | All double trials | |  |  |  | Response inhibition (proactive; indirect pathway) | All stop trials | |  |  |
|  | All double trials > all fixations | | |  |  |  | All stop trials > all double trials | | |  |
|  | All double trials > all ignore trials | | |  |  |  | All stop trials > all fixations | | |  |
|  | All double trials > all stop trials | | |  |  |  | All stop trials > all ignore trials | | |  |
|  | All double trials > correct stop signals | | |  |  |  | All stop trials > double fixations | | |  |
|  | All double trials > double fixations | | |  |  |  | All stop trials > double no-signals | | |  |
|  | All double trials > ignore fixations | | |  |  |  | All stop trials > double signals | | |  |
|  | All double trials > ignore no-signals | | |  |  |  | All stop trials > ignore fixations | | |  |
|  | All double trials > ignore signals | | |  |  |  | All stop trials > ignore no-signals | | |  |
|  | All double trials > incorrect stop signals | | |  |  |  | All stop trials > ignore signals | | |  |
|  | All double trials > stop fixations | | |  |  |  | Stop fixations | |  |  |
|  | All double trials > stop no-signals | | |  |  |  | Stop fixations > all double trials | | |  |
|  | All double trials > stop signals | | |  |  |  | Stop fixations > all ignore trials | | |  |
|  | All ignore trials | |  |  |  |  | Stop fixations > double fixations | | |  |
|  | All ignore trials > all fixations | | |  |  |  | Stop fixations > double no-signals | | |  |
|  | All ignore trials > all stop trials | | |  |  |  | Stop fixations > double signals | | |  |
|  | All ignore trials > correct stop signals | | |  |  |  | Stop fixations > ignore fixations | | |  |
|  | All ignore trials > double fixations | | |  |  |  | Stop fixations > ignore no-signals | | |  |
|  | All ignore trials > ignore fixations | | |  |  |  | Stop fixations > ignore signals | | |  |
|  | All ignore trials > incorrect stop signals | | |  |  |  | Incorrect stop signals | |  |  |
|  | All ignore trials > stop fixations | | |  |  |  | Incorrect stop signals > all double trials | | |  |
|  | All ignore trials > stop no-signals | | |  |  |  | Incorrect stop signals > all ignore trials | | |  |
|  | All ignore trials > stop signals | | |  |  |  | Incorrect stop signals > double fixations | | |  |
|  | Double signals | |  |  |  |  | Incorrect stop signals > double no-signals | | | |
|  | Double signals > all fixations | | |  |  |  | Incorrect stop signals > double signals | | |  |
|  | Double signals > all ignore trials | | |  |  |  | Incorrect stop signals > ignore fixations | | |  |
|  | Double signals > all stop trials | | |  |  |  | Incorrect stop signals > ignore no-signals | | | |
|  | Double signals > correct stop signals | | |  |  |  | Incorrect stop signals > ignore signals | | |  |
|  | Double signals > double fixations | | |  |  |  | Stop no-signals | |  |  |
|  | Double signals > double no-signals | | |  |  |  | Stop no-signals > all double trials | | |  |
|  | Double signals > ignore fixations | | |  |  |  | Stop no-signals > all fixations | | |  |
|  | Double signals > ignore no-signals | | |  |  |  | Stop no-signals > all ignore trials | | |  |
|  | Double signals > ignore signals | | |  |  |  | Stop no-signals > double fixations | | |  |
|  | Double signals > incorrect stop signals | | |  |  |  | Stop no-signals > double no-signals | | |  |
|  | Double signals > stop fixations | | |  |  |  | Stop no-signals > double signals | | |  |
|  | Double signals > stop no-signals | | |  |  |  | Stop no-signals > ignore fixations | | |  |
|  | Double signals > stop signals | | |  |  |  | Stop no-signals > ignore no-signals | | |  |
|  | Double no-signals | |  |  |  |  | Stop no-signals > ignore signals | | |  |
|  | Double no-signals > all fixations | | |  |  |  |  |  |  |  |
|  | Double no-signals > all ignore trials | | |  |  |  |  |  |  |  |
|  | Double no-signals > all stop trials | | |  |  |  |  |  |  |  |
|  | Double no-signals > correct stop signals | | |  |  |  |  |  |  |  |
|  | Double no-signals > double fixations | | |  |  |  |  |  |  |  |
|  | Double no-signals > ignore fixations | | |  |  |  |  |  |  |  |
|  | Double no-signals > incorrect stop signals | | | |  |  |  |  |  |  |
|  | Double no-signals > stop fixations | | |  |  |  |  |  |  |  |
|  | Double no-signals > stop no-signals | | |  |  |  |  |  |  |  |
|  | Double no-signals > stop signals | | |  |  |  |  |  |  |  |
|  | Ignore signals | |  |  |  |  |  |  |  |  |
|  | Ignore signals > all fixations | | |  |  |  |  |  |  |  |
|  | Ignore signals > all stop trials | | |  |  |  |  |  |  |  |
|  | Ignore signals > correct stop signals | | |  |  |  |  |  |  |  |
|  | Ignore signals > double fixations | | |  |  |  |  |  |  |  |
|  | Ignore signals > ignore fixations | | |  |  |  |  |  |  |  |
|  | Ignore signals > incorrect stop signals | | |  |  |  |  |  |  |  |
|  | Ignore signals > stop fixations | | |  |  |  |  |  |  |  |
|  | Ignore signals > stop no-signals | | |  |  |  |  |  |  |  |
|  | Ignore signals > stop signals | | |  |  |  |  |  |  |  |
|  | Ignore no-signals | |  |  |  |  |  |  |  |  |
|  | Ignore no-signals > all fixations | | |  |  |  |  |  |  |  |
|  | Ignore no-signals > all stop trials | | |  |  |  |  |  |  |  |
|  | Ignore no-signals > correct stop signals | | |  |  |  |  |  |  |  |
|  | Ignore no-signals > double fixations | | |  |  |  |  |  |  |  |
|  | Ignore no-signals > ignore fixations | | |  |  |  |  |  |  |  |
|  | Ignore no-signals > incorrect stop signals | | | |  |  |  |  |  |  |
|  | Ignore no-signals > stop fixations | | |  |  |  |  |  |  |  |
|  | Ignore no-signals > stop no-signals | | |  |  |  |  |  |  |  |
|  | Ignore no-signals > stop signals | | |  |  |  |  |  |  |  |
|  |  |  |  |  |  |  |  |  |  |  |

| Pathway categ. | Contrast |  |  |  |  | Pathway categ. | Contrast |  |  |  |
| --- | --- | --- | --- | --- | --- | --- | --- | --- | --- | --- |
| Response inhibition (reactive; hyperdirect pathway) | Stop signals |  |  |  |  | Unclear | All fixations |  |  |  |
|  | Stop signals > all double trials | | |  |  |  | All fixations > all double trials | | |  |
|  | Stop signals > all fixations | |  |  |  |  | All fixations > all ignore trials | | |  |
|  | Stop signals > all ignore trials | | |  |  |  | All fixations > all stop trials | | |  |
|  | Stop signals > double fixations | | |  |  |  | All fixations > correct stop signals | | |  |
|  | Stop signals > double no-signals | | |  |  |  | All fixations > double no-signals | | |  |
|  | Stop signals > double signals | | |  |  |  | All fixations > double signals | | |  |
|  | Stop signals > ignore fixations | | |  |  |  | All fixations > ignore no-signals | | |  |
|  | Stop signals > ignore no-signals | | |  |  |  | All fixations > ignore signals | | |  |
|  | Stop signals > ignore signals | | |  |  |  | All fixations > incorrect stop signals | | |  |
|  | Stop signals > stop fixations | | |  |  |  | All fixations > stop no-signals | | |  |
|  | Stop signals > stop no-signals | | |  |  |  | All fixations > stop signals | |  |  |
|  | Correct stop signals | |  |  |  |  | Double fixations | |  |  |
|  | Correct stop signals > all double trials | | |  |  |  | Double fixations > all double trials | | |  |
|  | Correct stop signals > all fixations | | |  |  |  | Double fixations > all ignore trials | | |  |
|  | Correct stop signals > all ignore trials | | |  |  |  | Double fixations > all stop trials | | |  |
|  | Correct stop signals > double fixations | | |  |  |  | Double fixations > correct stop signals | | |  |
|  | Correct stop signals > double no-signals | | |  |  |  | Double fixations > double no-signals | | |  |
|  | Correct stop signals > double signals | | |  |  |  | Double fixations > double signals | | |  |
|  | Correct stop signals > ignore fixations | | |  |  |  | Double fixations > ignore fixations | | |  |
|  | Correct stop signals > ignore no-signals | | |  |  |  | Double fixations > ignore no-signals | | |  |
|  | Correct stop signals > ignore signals | | |  |  |  | Double fixations > ignore signals | | |  |
|  | Correct stop signals > incorrect stop signals | | | |  |  | Double fixations > incorrect stop signals | | |  |
|  | Correct stop signals > stop fixations | | |  |  |  | Double fixations > stop fixations | | |  |
|  | Correct stop signals > stop no-signals | | |  |  |  | Double fixations > stop no-signals | | |  |
|  |  |  |  |  |  |  | Double fixations > stop signals | | |  |
|  |  |  |  |  |  |  | Ignore fixations | |  |  |
|  |  |  |  |  |  |  | Ignore fixations > all double trials | | |  |
|  |  |  |  |  |  |  | Ignore fixations > all ignore trials | | |  |
|  |  |  |  |  |  |  | Ignore fixations > all stop trials | | |  |
|  |  |  |  |  |  |  | Ignore fixations > correct stop signals | | |  |
|  |  |  |  |  |  |  | Ignore fixations > double fixations | | |  |
|  |  |  |  |  |  |  | Ignore fixations > double no-signals | | |  |
|  |  |  |  |  |  |  | Ignore fixations > double signals | | |  |
|  |  |  |  |  |  |  | Ignore fixations > ignore no-signals | | |  |
|  |  |  |  |  |  |  | Ignore fixations > ignore signals | | |  |
|  |  |  |  |  |  |  | Ignore fixations > incorrect stop signals | | |  |
|  |  |  |  |  |  |  | Ignore fixations > stop fixations | | |  |
|  |  |  |  |  |  |  | Ignore fixations > stop no-signals | | |  |
|  |  |  |  |  |  |  | Ignore fixations > stop signals | | |  |
|  |  |  |  |  |  |  | Stop fixations > all stop trials | | |  |
|  |  |  |  |  |  |  | Stop fixations > correct stop signals | | |  |
|  |  |  |  |  |  |  | Stop fixations > incorrect stop signals | | |  |
|  |  |  |  |  |  |  | Stop fixations > stop no-signals | | |  |
|  |  |  |  |  |  |  | Stop fixations > stop signals | | |  |
|  |  |  |  |  |  |  | All ignore trials > all double trials | | |  |
|  |  |  |  |  |  |  | All ignore trials > double no-signals | | |  |
|  |  |  |  |  |  |  | All ignore trials > double signals | | |  |
|  |  |  |  |  |  |  | All stop trials > stop fixations | | |  |
|  |  |  |  |  |  |  | Double no-signals > double signals | | |  |
|  |  |  |  |  |  |  | Double no-signals > ignore no-signals | | |  |
|  |  |  |  |  |  |  | Double no-signals > ignore signals | | |  |
|  |  |  |  |  |  |  | Ignore no-signals > all double trials | | |  |
|  |  |  |  |  |  |  | Ignore no-signals > double no-signals | | |  |
|  |  |  |  |  |  |  | Ignore no-signals > double signals | | |  |
|  |  |  |  |  |  |  | Ignore no-signals > ignore signals | | |  |
|  |  |  |  |  |  |  | Ignore signals > all double trials | | |  |
|  |  |  |  |  |  |  | Ignore signals > double no-signals | | |  |
|  |  |  |  |  |  |  | Ignore signals > double signals | | |  |
|  |  |  |  |  |  |  | Ignore signals > ignore no-signals | | |  |
|  |  |  |  |  |  |  | Stop no-signals > correct stop signals | | |  |
|  |  |  |  |  |  |  | Stop no-signals > incorrect stop signals | | |  |
|  |  |  |  |  |  |  | Stop no-signals > stop fixations | | |  |
|  |  |  |  |  |  |  | Stop no-signals > stop signals | | |  |
|  |  |  |  |  |  |  | Incorrect stop signals > all fixations | | |  |
|  |  |  |  |  |  |  | Incorrect stop signals > correct stop signals | | | |
|  |  |  |  |  |  |  | Incorrect stop signals > stop fixations | | |  |
|  |  |  |  |  |  |  | Incorrect stop signals > stop no-signals | | |  |
|  |  |  |  |  |  |  |  |  |  |  |
|  |  |  |  |  |  |  |  |  |  |  |

**SI table 1. All contrasts computed for the compound contrast analyses.**

199 separate contrast combinations were categorised Pathway categ.) as reflective of either the direct, the indirect or the hyperdirect pathway or as unclear. All fixations = all fixations that were presented in each of the task contexts, stop fixations = fixations that were presented in the stop context, double fixations = fixations that were presented in the double-response task, ignore fixations = fixations presented in the ignore task, all stop trials = all trials presented in the stop task regardless of whether signals or no-signal trials, all double trials = all trials presented in the double-response task regardless of whether signals or no-signal trials, all ignore trials = all trials presented in the ignore task, signals = signal trials presented in a specific context, no-signals = no-signal trials presented in a specific context, correct stop signals = stop signal trials that were successfully inhibited, incorrect stop signals = stop signal trials that were responded to (i.e. an incorrect response).

**1.7 Temporal signal to noise maps**

Temporal signal to noise maps (tSNR) were calculated to asses differences in signal to noise across different regions. For each run of fMRI data this involved dividing the mean across the time series by the standard deviation across the time series for the non-preprocessed EPI data (Murphy et al., 2007). Averages were then drawn across runs and then across participants to produce group means. Masks used in primary analyses (see 2.7.3) were used to isolate tSNR estimates for each ROI inverted to spatial coordinates for each tSNR map using FSLs’ FLIRT. Analysis scripts to calculate participant’s tSNR maps are available at <https://osf.io/3b8yp/> and group means are depicted below.

# **Analysis and results**

## Behavioural results summary

The analyses of this section and those reported in the manuscript confirmed that participants performed within pre-specified benchmarks (see <https://osf.io/zqefx/>, section 4.2.3). These include assessment of participant’s accuracy in performing the tasks (SI section 2.1.1) and reaction times (RTs; SI section 2.1.2). Further analyses were conducted to explore task specific measures related to the SST (SI section 2.1.3.1) and the DT (SI section 2.1.3.2).

Note, that a pre-registered criterion was that behavioural analyses (only) would be conducted with RTs less than 150ms excluded as these would be considered anticipatory responses. However, all data was included in the imaging based analyses (as pre-registered) to maintain identical stimuli across each condition. Frequentist analyses were conducted using SPSS (IBM Corp (2015), version 23) and Bayes Factors (BFs) were computed using JASP (JASP Team (2019), version 0.9.2.0; using default priors). Where relevant the Holm-Bonferonni method (Aickin and Gensler, 1996) was used to correct for multiple comparisons and the α-level for comparison is denoted as the *p*-value subscript (or explicitly within a table) where necessary.

## Accuracy

Participants performed the behavioural tasks in line with, and in excess of, the levels to which they were trained. Accuracy rates including trials with RTs <150ms are reported in the manuscript, those with RTs <150ms excluded are reported in SI table 2. Accuracy was greater than 85% for signal and no-signal trials in the IT and DT, and for no-signal trials in the SST. Further, accuracy for signal trials in the SST was around the target value of 50% successful stopping, as described in the pre-registered document. The maintenance of ~50% successful stopping validates the method of online updating of the stop signal delay (SSD) according to the fitting of response inhibition functions to each pair of runs data (group mean = adjusted R^2^=0.96, *SD*=0.06; see section 4.2.3 of <https://osf.io/zqefx/> and clarification section 1 of <https://osf.io/27gmh/>).

|  | Excluding RTs<150ms | |
| --- | --- | --- |
|  | % accuracy | SD |
| Ignore no-signal | 94.97 | 3.13 |
| Ignore signal | 95.51 | 3.31 |
| Double no-signal | 96.76 | 2.03 |
| Double signal | 93.12 | 3.55 |
| Stop no-signal | 98.51 | 1.32 |
| Stop signal | 45.45 | 6.03 |

**SI table 2. Accuracy rates across different trial types.**

RTs = reaction times; SD = standard deviation.

Repeated measures ANOVA of accuracy rates excluding responses <150ms were in line with those reported in the main manuscript (section 3.1). That is, main effects of context (stop, double, ignore: F_(2,58)_=1245.62, *p*<0.001, BF=8.41×10^12^) and trial types (signal, no-signal: F_(1,29)_=2471.73, *p*<0.001, BF=2.39×10^9^) were found, as was an interaction effect (F_(1.44,41.84)_=1852.12, *p*<0.001 (degrees of freedom Greenhouse-Geisser corrected), BF=3.93×10^105^). No-signal trial performance significantly differed across all contexts (SST > DT > IT, all *p*<0.001, BF>211.77). Within contexts, signal and no-signal trial accuracy rates differed for the SST (*p_0.0167_*<0.001, BF=2.01×10^27^) and DT (*p_0.025_*<0.001, BF=23406.88), but not for the IT (*p_0.05_*=0.115, BF=0.63).

## Reaction times

Reaction times (RTs) were computed for correct trials across all conditions, with the exception of stop signal trials where any response (although incorrect) was considered. RTs (SI table 3) were in line with those previously reported for comparable tasks (e.g. Verbruggen et al., 2010).

|  | Excluding RTs<150ms | |
| --- | --- | --- |
|  | RT (ms) | SD |
| Ignore no-signal | 386.79 | 41.10 |
| Ignore signal | 397.47 | 44.22 |
| Double no-signal | 415.71 | 39.87 |
| Double signal (initial response) | 422.04 | 43.58 |
| Stop no-signal | 457.10 | 58.63 |
| Stop signal (unsuccessful) | 427.27 | 56.02 |

**SI table 3. Accuracy rates across different trial types.**

RT = reaction time in ms; SD = standard deviation; RTs for stop signal trials are those where a response was made on signal trials (i.e. an unsuccessful stop).

Repeated measures ANOVA of RTs excluding responses <150ms were in line with those reported in the main manuscript (section 3.1). That is, main effects of context (stop, double, ignore: F_(1.44,41.67)_=43.42, *p*<0.001, BF=2.33×10^20^) and trial types (signal, no-signal: F_(1,29)_=6.53, *p*=0.016, BF=0.24) were found, as was an interaction effect (F_(1.65,47.97)_=151.47, *p*<0.001, BF=2.24×10^5^). Degrees of freedom Greenhouse-Geisser corrected. Pairwise comparisons demonstrated longer RTs to no-signal trials in the SST followed by the DT and IT (all *p*<0.001, all BF>12397.95). Further RTs to signal trials were longer than no-signal trials in the DT (*p_0.05_*=0.007, BF=6.40) and IT (*p_0.05_*<0.001, BF=111866.55), and shorted in the SST (*p*_0.0167_<0.001, BF=5.76×10^8^).

## Task specific measures

In the SST and DT, specific variables were computed to explore the relationship between task performance and BOLD activity in specific ROIs (see SI section 2.3). The calculation of these are outlined below.

## Stop-signal task

In the SST, the latency of the stop process (stop signal reaction time; SSRT) is estimated, here, using the mean and integration methods (Logan and Cowan, 1984). Data were initially screened to ensure the assumptions underlying these approaches were met:

1. As discussed above, successful inhibition occurred on ~50% of stop signal trials (SI table 2), ensuring optimum competition between stop and go processes on stop signal trials (Logan and Cowan, 1984).
2. Mean RTs on signal trials (where participants failed to inhibit their response) was shorter than on no-signal trials in accord with the assumptions of the independent race model (Logan & Cowan, 1984). Inclusive of RTs<150ms: t_(29)_=10.36, *p*_0.0167_<0.001, BF=3.20×10^8^. Excluding RTs<150ms: (t_(29)_=10.64, *p*_0.0167_<0.001, BF=5.76×10^8^).

Group level estimates of SSRT are presented in SI table 4. SSRT estimates were computed using the mean and integration methods (Logan and Cowan, 1984) across every 2 fMRI runs (with the exception of excluded runs due to excess motion) as SSDs were matched across 2 successive runs before adjustment (see section 5.3 here: <https://osf.io/zqefx/> and clarification point 1 here: <https://osf.io/27gmh/>) and averaged across the session. SSRTs calculated using the mean method involved subtracting the SSD at which participants correctly inhibit their response on 50% trials from their mean RT on no-signal (go) trials in the SST (where all RTs with the exception of missed responses (0ms RTs) were considered). Here, we made use of the 50%SSD that was used to set all other SSDs every 2^nd^ fMRI run. Directional choice errors were not exclude form RT measures. The mean 50% SSD across participants was 246.67ms (± SD 60.70ms). SSRTs calculated using the integration method also made use of this 50%SSD, with the probability of responding to signal trials (P(respond|signal)) taken across *all* SSDs. This differs from the typical approach of calculating SSRT separately for each SSD. This was because there were only 6 instances of each of the 6 SSDs presented across 2 fMRI runs, rendering the typical approach unreliable. Table SI 4 summarises SSRT estimations using the mean method, the integration method and the integration method with replacement, in which failed Go responses are replaced by maximum RTs drawn from each participants SST performance (see Verbruggen et al., 2019).

|  | Including  RTs <150ms | |  | | Excluding  RTs <150ms | | |  |
| --- | --- | --- | --- | --- | --- | --- | --- | --- |
|  | SSRT (ms) | SD | |  | | SSRT (ms) | SD | |
| Mean method | 231.88 | 37.41 | |  | | 232.23 | 37.16 | |
| Integration method  Integration method with replacement | 228.66  228.91 | 31.95  31.97 | |  | | 228.36  232.12 | 32.24  32.24 | |

**SI table 4. Estimates of stop signal reaction time**

SSRT = stop signal reaction time in ms; SD = standard deviation. Estimates were produced using the mean, integration and integration method where 0 RTs are replaced by maximum RTs (Verbruggen et al., 2019). Note that RTs <150ms were only excluded from go RT distributions. All responses to stop signal trials were considered incorrect.

## Double response task

In the DT we were interested in locating the central decision bottleneck or the psychological refractory period (PRP) for brain-behaviour analyses (SI section 2.3.2.2 and 2.3.2.3). The PRP is a phenomenon observed in dual-task situations where RT to a second stimulus is prolonged when presented in close temporal proximity to a first (e.g. Pashler, 1994; Pashler and Johnston, 1989; Ruthruff et al., 2003; Telford, 1931). Here, the locus of the central bottleneck was estimated individually for each participant using the quantification procedure outlined in <https://osf.io/zqefx/>, section 6.1. Briefly, this involved applying a series of pairs of weighted linear fits to RTs over the range stimulus onset asynchronies (SOAs) between the stimulus and the signal. The PRP was quantified using the area between these the two fits which most closely resembled the data, and the start of the PRP was the intercept between the two fits (see <https://osf.io/zqefx/>, figure 6). Missed additional responses and instances of negative DRT2s (i.e. where the 2^nd^ response is made before the signal onset) were excluded from these analyses. The analyses were conducted irrespective of RTs<150ms since the 2^nd^ (additional) response on signal trials was used for the computation. The size of the PRP (area) was quantified to estimate the magnitude of slowing caused by the central bottleneck.

- The mean locus of the PRP across participants was identified at an SOA of 190.31ms (SD = 74.84ms; SI figure 1) and the quality of fits were good (group mean adjusted R^2^=0.92 ± 0.10).
- As expected, the largest DRT2 was found at the shortest SOA (F_(1.33,38.49)_=115.96, *p*_(0.05)_<0.001, Greenhouse-Geisser corrected, BF=5.12×10^18^) and was reliably longer than DRT2 at the intercept and DRT2 at the longest SOA. DRT2 at the longest SOA was also found to be greater than DRT2 at the intercept (all *p*<0.002, all BF>18.22).
- The increase in DRT2 between the intercept and largest SOA was unexpected (SI figure 1). Greater variability in DRT2 was also found at the longest SOA relative to that at the intercept SOA (longest SOA: 476.63ms ± 57.53ms; intercept SOA: 484.13ms ± 48.49ms). Speculatively, this relative increase in DRT2 at the latest SOA may be due to a reduction in preparation to execute an additional response with increased SOA. Alternatively, it could be an artefact of the fitting procedure resulting from the greater variability at longer SOAs.
- The size of the PRP (area) across participants was 0.01 (*SD* = 0.006).

**
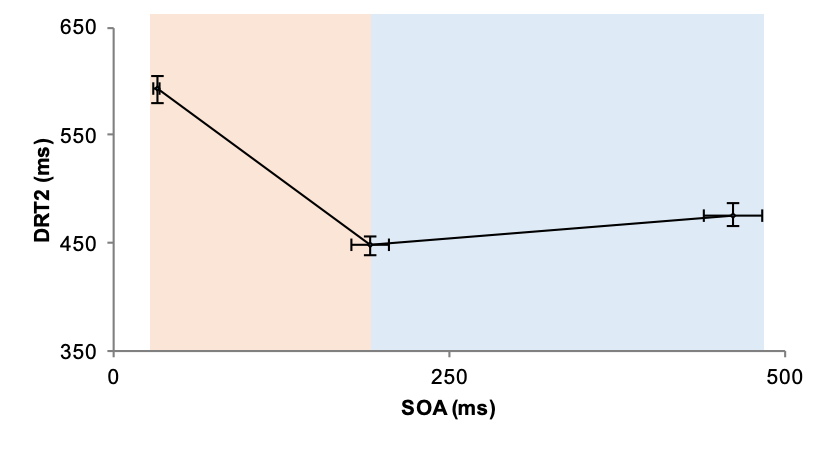
**

**SI figure 1. Mean psychological refractory period across participants.**

The time to execute the second response relative to the signal onset (DRT2) was found to decrease as the delay between stimulus and signal onsets (SOA) increased during the pre-bottleneck stage (peach area), relative to the post-bottleneck stage (blue area). Pre- and post-bottleneck stages were divided according to the point of intercept between weighted linear fits and the mean RT on no-signal trials in the DT. Error bars are ± 1 standard error.

## Common and distinct cortical processes under different action-updating contexts: pre-registered and exploratory analyses

Pre-registered contrast analyses are reported in SI table 5 and pre-registered conjunction and disjunction analyses are reported in SI figure 2. These are in addition to and consistent with those reported in the main text. Detail relating to %BOLD and cluster based statistics for the exclusive and general recruitment of cortical ROIs are reported in SI table 6.


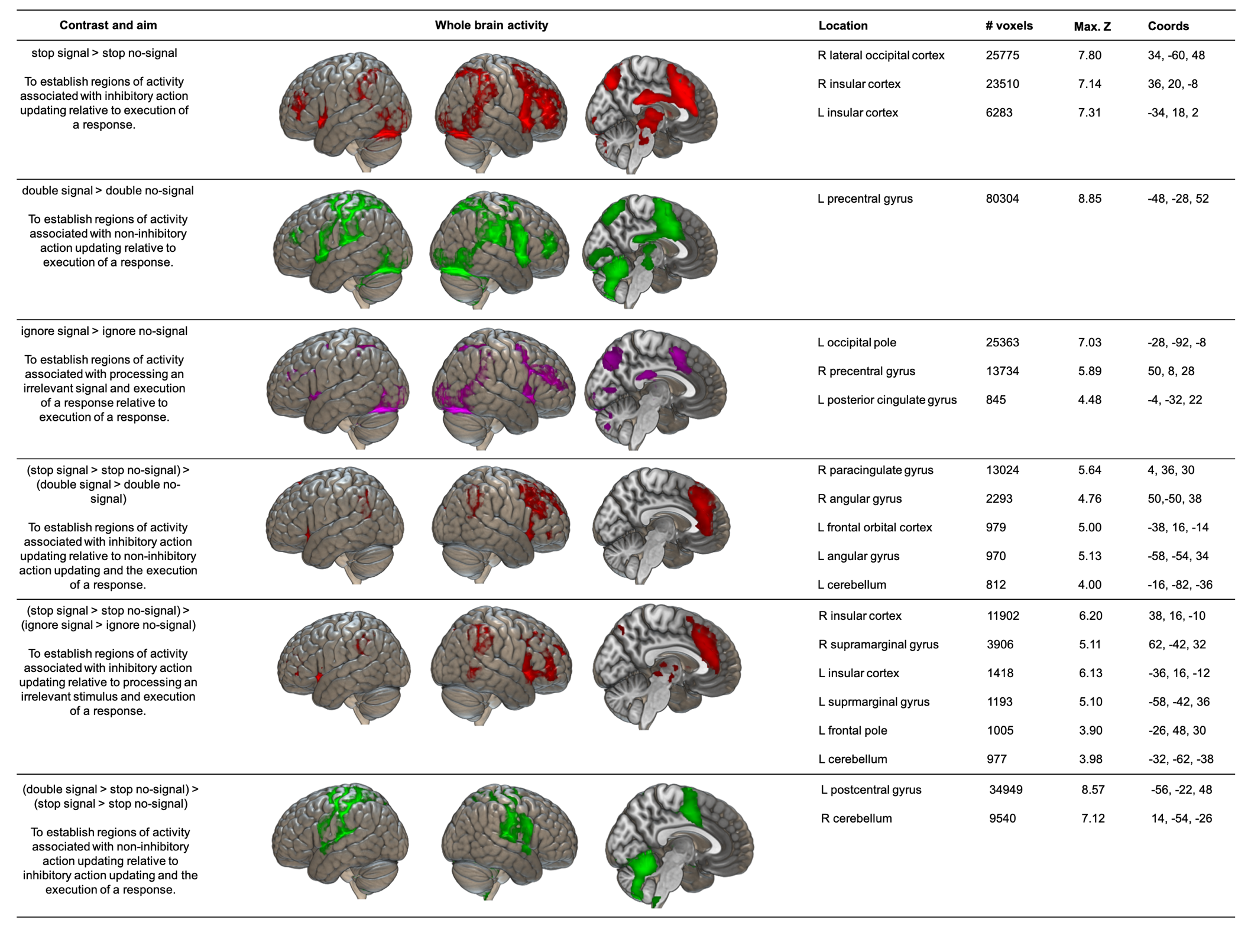


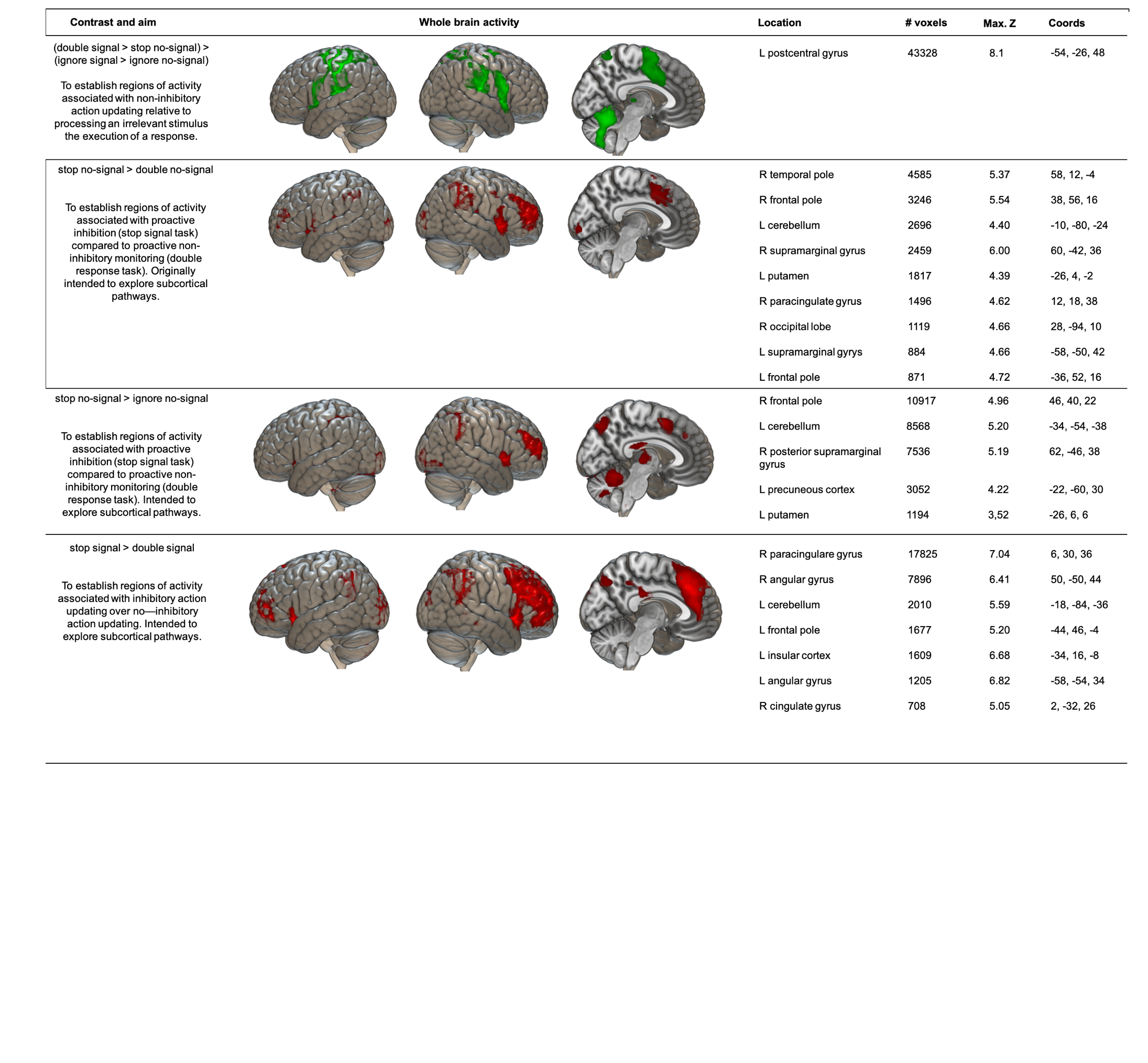


**
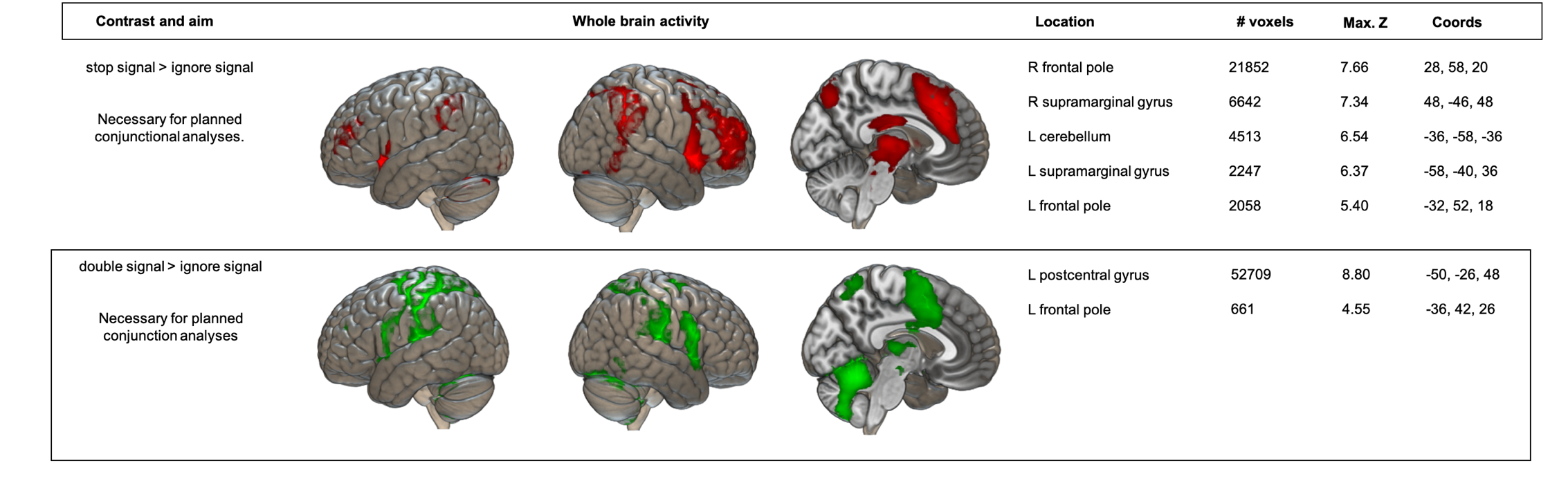
**

**SI table 5. Pre-registered contrasts.**

Results of cluster based analysis of pre-registered contrasts. Significant clusters are depicted in colour (Z>2.3, *p*<0.05). The aim and rationale behind each of the contrasts is outlined as are details of the significant clusters yielded. Red activity = activity associated with response inhibition; green = activity associated with non-inhibitory action updating; violet = activity associated with responding to a signal on ignore signal trials. Location = labels as per Harvard-Oxford Cortical and Subcortical atlases (Makris, Goldstein, Kennedy et al., 2006; Frazier, Chiu, Breeze et al., 2005; Desikan, Ségonne, Fischl et al., 2006; Goldstein, Seidman, Makris et al., 2007) for illustration purposes; # voxels= number of voxels comprising each of the significant clusters; Max Z= maximum Z-value within clusters; Coords= coordinates of maximum Z-value within each cluster. Images presented in neurological format (L=L; R=R) in MNI space.


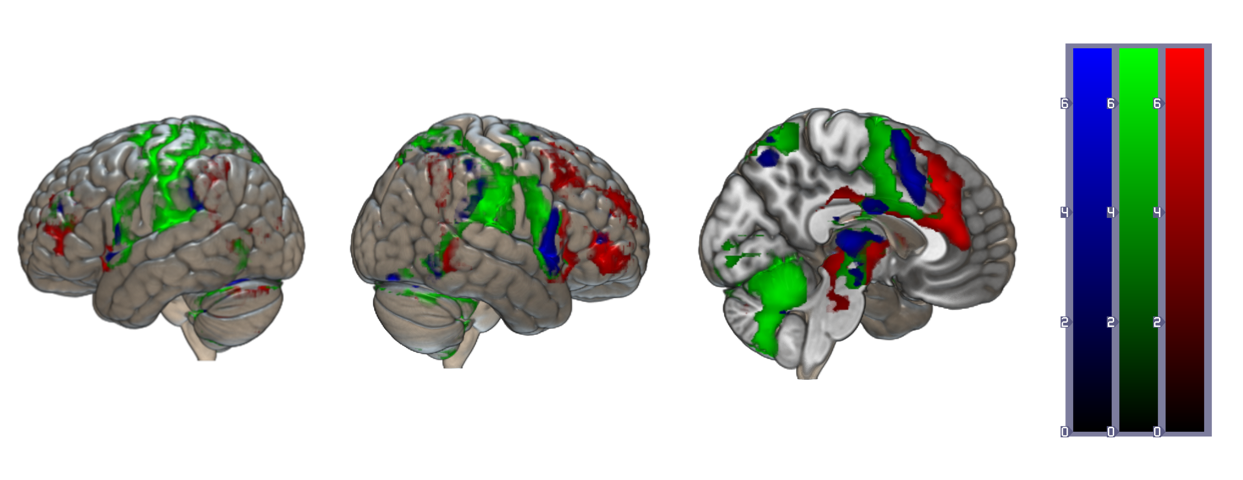


**SI figure 2. Pre-registered conjunction and disjunction analyses.**

Pre-registered conjunction and disjunction analyses not reported in the manuscript. Cluster based activity significant at Z>2.3, *p*<0.05. Images are illustrated in neurological format (L=L; R=R). Red regions = activity unique to inhibitory action updating (stop signal > stop no-signal) NOT (ignore signal > ignore no-signal); green regions = activity unique to non-inhibitory action updating (double signal > double no-signal) NOT (ignore signal > ignore no-signal); blue regions = activity common to both types of updating (stop signal > ignore signal) ∩ (double signal > ignore signal). Scale corresponds to Z-statistic values.

| Analysis |  |  | ROI | # | % | Z | MNI |
| --- | --- | --- | --- | --- | --- | --- | --- |
| **Exclusive inhibitory** | |  | right IFG | 501 | 40.02 | 6.00 | 22,71,51 |
| (stop signal > stop no-signal) NOT | | | Pars op | 102 | 14.74 | 6.00 | 22,71,51 |
| (double signal > double no-signal) | | | Pars tri | 343 | 68.33 | 5.51 | 19,80,45 |
|  |  |  | pre-SMA | 385 | 16.14 | 6.49 | 44,73,60 |
| **Exclusive non-inhibitory** | | | right IFG | 0 | N/A | N/A | N/A |
| (double signal > double no-signal) NOT | | | Pars op | 0 | N/A | N/A | N/A |
| (stop signal > stop no-signal) | | | Pars tri | 0 | N/A | N/A | N/A |
|  |  |  | pre-SMA | 372 | 15.59 | 6.25 | 47,64,60 |
| **General updating** | |  | right IFG | 641 | 51.20 | 6.04 | 19,68,47 |
| (stop signal > stop no-signal) AND | | | Pars op | 590 | 85.26 | 6.04 | 19,68,47 |
| (double signal > double no-signal) | | | Pars tri | 49 | 9.76 | 3.75 | 24,79,44 |
|  |  |  | pre-SMA | 654 | 27.41 | 5.29 | 45,71,57 |
| **Exclusive inhibitory** | |  | right IFG | 207 | 16.53 | 5.08 | 19,71,40 |
| (stop signal > stop no-signal) NOT | | | Pars op | 72 | 10.40 | 5.08 | 19,71,40 |
| (ignore signal > ignore no-signal) | | | Pars tri | 127 | 25.30 | 4.97 | 20,74,34 |
|  |  |  | pre-SMA | 573 | 24.02 | 6.00 | 42,74,60 |
| **Exclusive non-inhibitory** | | | right IFG | 37 | 2.96 | 6.61 | 15,69,45 |
| (double signal > double no-signal) NOT | | | Pars op | 34 | 4.91 | 6.61 | 15,69,45 |
| (ignore signal > ignore no-signal) | | | Pars tri | 2 | 0.40 | 2.95 | 19,74,33 |
|  |  |  | pre-SMA | 517 | 21.67 | 6.25 | 47,64,60 |
| **General updating** | |  | right IFG | 299 | 23.88 | 5.26 | 18,68,41 |
| (stop signal > ignore signal) AND | | | Pars op | 299 | 43.21 | 5.26 | 18,68,41 |
| (double signal > ignore signal) | | | Pars tri | 0 | N/A | N/A | N/A |
|  |  |  | pre-SMA | 479 | 20.08 | 4.69 | 44,69,58 |

**SI table 6. Exclusive and general recruitment of cortical ROIs.**

Summary of clusters (Z>2.3, *p*<.05) within the right inferior frontal gyrus (right IFG) and pre-supplementary motor area (pre-SMA) associated with disjunction and conjunction analyses. ROI = region of interest; # = number of activated voxels within the ROI; % = percent of ROI activated; Z = maximum Z-value within ROI; MNI = MNI coordinates corresponding to the maximum Z-value; NOT = refers to the logical not rather than a subtraction; AND = conjunction. Note that exploration of the subdivisions of the right IFG, the *pars opercularis* (pars op) and the *pars triangularis* (pars tri), is in addition to pre-registered ROIs.

## Brain-behaviour relationships

The results for pre-registered brain-behaviour analyses are reported separately for the SST (SI section 2.3.1) and DT (SI section 2.3.2). Contrasts included in these secondary analyses were conducted in general linear models (GLMs) separate to those presented in SI table 5 and in the manuscript. Pre-registered hypotheses relating to these analyses can be found in section 3.2 of the pre-registration document (<https://osf.io/zqefx/>).

## Stop signal task

## Correlation between %BOLD and SSRT

Correlation analyses were conducted between SSRT and %BOLD extracted from cortical and subcortical ROIs from the contrast stop signal > stop no-signal. Analyses were conducted separately for SSRTs calculated using the mean method (SI table 7), the integration method (SI table 8) and the integration method where failed Go RT’s are replace by maximum RT values (SI table 9). Partial correlation analyses were conducted to control for the effect of proactive slowing (calculated as the difference between mean RTs on no-signal trials in the SST and IT). Note that BFs were not calculated for partial correlations (SI tables 7, 8 and 9). Correlation analyses for %BOLD and proactive slowing alone (SI table 10) were also conducted.

We expected activity in regions crucial for response inhibition to be negatively correlated with SSRT. This was because shorter SSRTs are associated with better inhibitory control. However, correlations did not clearly support such a relationship:

- Although significant correlations between %BOLD in the left SN (defined manually or via the ATAG atlas) and mean SSRT were identified, these did not survive correction for multiple comparisons (SI table 7).
- No significant correlations between %BOLD and mean SSRT were found when proactive slowing was controlled for (SI table 7).
- No significant correlations between %BOLD and integration SSRT were found irrespective of whether or not proactive slowing was controlled for or whether or not the integration method involved replacement of failed go RTs (SI table 8 and 9).
- No significant correlations between %BOLD and proactive slowing was identified (SI table 10).

|  |  | Mean SSRT | | | Proactive slowing | |
| --- | --- | --- | --- | --- | --- | --- |
| Hem | ROI | r | *p* | BF | r | *p* |
| Right | IFG | -0.07 | 0.72 | 0.15 | -0.04 | 0.82 |
|  | pre-SMA | 0.17 | 0.36 | 0.21 | 0.23 | 0.23 |
|  | STR | 0.24 | 0.19 | 0.33 | 0.22 | 0.24 |
|  | GPe | 0.30 | 0.10 | 0.53 | 0.28 | 0.14 |
|  | GPi | 0.30 | 0.11 | 0.50 | 0.28 | 0.14 |
|  | Atlas STN | 0.17 | 0.36 | 0.21 | 0.17 | 0.39 |
|  | Atlas SN | 0.43 | 0.02 | 2.17 | 0.44 | 0.02 |
|  | THAL | 0.23 | 0.21 | 0.31 | 0.24 | 0.22 |
|  | Manual STN | 0.08 | 0.69 | 0.15 | 0.07 | 0.71 |
|  | Manual SN | 0.21 | 0.26 | 0.26 | 0.22 | 0.25 |
| Left | IFG | 0.20 | 0.29 | 0.25 | 0.16 | 0.40 |
|  | pre-SMA | 0.20 | 0.28 | 0.25 | 0.27 | 0.16 |
|  | STR | 0.34 | 0.06 | 0.77 | 0.30 | 0.11 |
|  | GPe | 0.31 | 0.09 | 0.59 | 0.28 | 0.14 |
|  | GPi | 0.07 | 0.70 | 0.15 | 0.08 | 0.67 |
|  | Atlas STN | 0.06 | 0.77 | 0.15 | 0.05 | 0.81 |
|  | Atlas SN | 0.21 | 0.27 | 0.26 | 0.22 | 0.26 |
|  | THAL | 0.31 | 0.10 | 0.54 | 0.28 | 0.14 |
|  | Manual STN | 0.17 | 0.36 | 0.21 | 0.17 | 0.39 |
|  | Manual SN | 0.43 | 0.02 | 2.17 | 0.44 | 0.02 |

**SI table 7. Pearson’s correlation analyses between %BOLD and SSRT estimated using the mean method.**

SSRT was calculated using the mean method. Additional analyses included proactive slowing as a covariate. Hem= hemisphere; ROI = region of interest; r = Pearson’s correlation; *p* = p-value; α = alpha for comparison; BF = Bayes factor; IFG = inferior frontal gyrus; pre-SMA = pre-supplementary motor area; STR = striatum; GPe = globus pallidus externa; GPi = globus pallidus interna; Atlas STN = subthalamic nucleus defined by the ATAG atlas; Atlas SN =substantia nigra defined by the ATAG atlas; THAL = thalamus; Manual STN = subthalamic nucleus defined manually; Manual SN = substantia nigra defined by the ATAG atlas but with voxels identified as STN (manually) excluded. No BFs>3 were found. *p*<0.05 failed to survive correction for multiple comparisons (α=0.0025). All degrees of freedom=28 for correlation analyses and 27 for partial correlation analyses.

|  |  | Integration SSRT | | | Proactive slowing | |
| --- | --- | --- | --- | --- | --- | --- |
| Hem | ROI | r | *p* | BF | r | *p* |
| Right | IFG | -0.07 | 0.71 | 0.15 | -0.04 | 0.84 |
|  | pre-SMA | 0.16 | 0.41 | 0.20 | 0.23 | 0.22 |
|  | STR | 0.22 | 0.25 | 0.27 | 0.19 | 0.32 |
|  | GPe | 0.23 | 0.22 | 0.30 | 0.20 | 0.30 |
|  | GPi | 0.25 | 0.18 | 0.34 | 0.22 | 0.25 |
|  | Atlas STN | 0.02 | 0.92 | 0.14 | 0.01 | 0.97 |
|  | Atlas SN | 0.30 | 0.11 | 0.52 | 0.31 | 0.10 |
|  | THAL | 0.18 | 0.34 | 0.22 | 0.18 | 0.35 |
|  | Manual STN | -0.01 | 0.96 | 0.14 | -0.02 | 0.92 |
|  | Manual SN | 0.14 | 0.45 | 0.19 | 0.15 | 0.43 |
| Left | IFG | 0.20 | 0.29 | 0.25 | 0.15 | 0.44 |
|  | pre-SMA | 0.15 | 0.42 | 0.20 | 0.24 | 0.21 |
|  | STR | 0.32 | 0.08 | 0.64 | 0.27 | 0.16 |
|  | GPe | 0.30 | 0.10 | 0.52 | 0.26 | 0.17 |
|  | GPi | 0.07 | 0.70 | 0.15 | 0.09 | 0.66 |
|  | Atlas STN | -0.05 | 0.79 | 0.15 | -0.07 | 0.73 |
|  | Atlas SN | 0.14 | 0.46 | 0.18 | 0.15 | 0.44 |
|  | THAL | 0.27 | 0.15 | 0.40 | 0.23 | 0.23 |
|  | Manual STN | 0.02 | 0.92 | 0.14 | 0.01 | 0.97 |
|  | Manual SN | 0.30 | 0.11 | 0.52 | 0.31 | 0.10 |

**SI table 8. Pearson’s correlation analyses between %BOLD and SSRT estimated using the integration method.**

SSRT was calculated using the integration method. Additional analyses included proactive slowing as a covariate. Hem= hemisphere; ROI = region of interest; r = Pearson’s correlation; *p* = p-value; α = alpha for comparison; BF = Bayes factor; IFG = inferior frontal gyrus; pre-SMA = pre-supplementary motor area; STR = striatum; GPe = globus pallidus externa; GPi = globus pallidus interna; Atlas STN = subthalamic nucleus defined by the ATAG atlas; Atlas SN =substantia nigra defined by the ATAG atlas; THAL = thalamus; Manual STN = subthalamic nucleus defined manually; Manual SN = substantia nigra defined by the ATAG atlas but with voxels identified as STN (manually) excluded. No *p*<0.05 or BFs>3 were found. All degrees of freedom=28 for correlation analyses and 27 for partial correlation analyses.

|  |  | Integration SSRT with replacement | | | Proactive slowing | |
| --- | --- | --- | --- | --- | --- | --- |
| Hem | ROI | r | *p* | BF | r | *p* |
| Right | IFG | -0.07 | 0.70 | 0.15 | -0.04 | 0.84 |
|  | pre-SMA | 0.16 | 0.41 | 0.20 | 0.23 | 0.22 |
|  | STR | 0.21 | 0.26 | 0.27 | 0.19 | 0.32 |
|  | GPe | 0.23 | 0.22 | 0.29 | 0.20 | 0.30 |
|  | GPi | 0.25 | 0.19 | 0.34 | 0.22 | 0.25 |
|  | Atlas STN | 0.02 | 0.93 | 0.14 | 0.01 | 0.97 |
|  | Atlas SN | 0.30 | 0.11 | 0.51 | 0.31 | 0.10 |
|  | THAL | 0.18 | 0.35 | 0.22 | 0.18 | 0.35 |
|  | Manual STN | -0.01 | 0.95 | 0.14 | -0.02 | 0.92 |
|  | Manual SN | 0.14 | 0.46 | 0.18 | 0.15 | 0.43 |
| Left | IFG | 0.20 | 0.30 | 0.24 | 0.15 | 0.44 |
|  | pre-SMA | 0.15 | 0.43 | 0.19 | 0.24 | 0.21 |
|  | STR | 0.32 | 0.08 | 0.63 | 0.27 | 0.16 |
|  | GPe | 0.30 | 0.11 | 0.52 | 0.26 | 0.17 |
|  | GPi | 0.08 | 0.69 | 0.15 | 0.09 | 0.66 |
|  | Atlas STN | -0.05 | 0.79 | 0.15 | -0.07 | 0.73 |
|  | Atlas SN | 0.14 | 0.47 | 0.18 | 0.15 | 0.44 |
|  | THAL | 0.27 | 0.15 | 0.39 | 0.23 | 0.23 |
|  | Manual STN | 0.02 | 0.93 | 0.14 | 0.01 | 0.97 |
|  | Manual SN | 0.30 | 0.11 | 0.51 | 0.31 | 0.10 |

**SI table 9. Pearson’s correlation analyses between %BOLD and SSRT estimated using the integration method with replacement.**

SSRT was calculated using the integration method with replacement where failed go RTs are replace by participant maximum RTs rather than excluded. Additional analyses included proactive slowing as a covariate. Hem= hemisphere; ROI = region of interest; r = Pearson’s correlation; *p* = p-value; α = alpha for comparison; BF = Bayes factor; IFG = inferior frontal gyrus; pre-SMA = pre-supplementary motor area; STR = striatum; GPe = globus pallidus externa; GPi = globus pallidus interna; Atlas STN = subthalamic nucleus defined by the ATAG atlas; Atlas SN =substantia nigra defined by the ATAG atlas; THAL = thalamus; Manual STN = subthalamic nucleus defined manually; Manual SN = substantia nigra defined by the ATAG atlas but with voxels identified as STN (manually) excluded. No *p*<0.05 or BFs>3 were found. All degrees of freedom=28 for correlation analyses and 27 for partial correlation analyses.

| Proactive slowing | | | | |
| --- | --- | --- | --- | --- |
| Hem | ROI | r | *p* | BF |
| Right | IFG | 0.11 | 0.56 | 0.17 |
|  | pre-SMA | 0.19 | 0.30 | 0.24 |
|  | STR | -0.12 | 0.54 | 0.17 |
|  | GPe | -0.14 | 0.48 | 0.18 |
|  | GPi | -0.13 | 0.50 | 0.18 |
|  | Atlas STN | -0.04 | 0.83 | 0.14 |
|  | Atlas SN | -0.01 | 0.97 | 0.14 |
|  | THAL | -0.03 | 0.89 | 0.14 |
|  | Manual STN | -0.03 | 0.87 | 0.14 |
|  | Manual SN | 0.01 | 0.97 | 0.14 |
| Left | IFG | -0.19 | 0.31 | 0.23 |
|  | pre-SMA | 0.22 | 0.25 | 0.28 |
|  | STR | -0.24 | 0.20 | 0.33 |
|  | GPe | -0.19 | 0.31 | 0.24 |
|  | GPi | 0.03 | 0.90 | 0.14 |
|  | Atlas STN | -0.04 | 0.83 | 0.14 |
|  | Atlas SN | 0.01 | 0.96 | 0.14 |
|  | THAL | -0.17 | 0.37 | 0.21 |
|  | Manual STN | -0.04 | 0.83 | 0.14 |
|  | Manual SN | -0.01 | 0.97 | 0.14 |

**SI table 10. Pearson’s correlation analyses between %BOLD and proactive slowing.**

Proactive slowing was calculated as the difference in RTs to no-signal trials in the SST and IT. Hem= hemisphere; ROI = region of interest; r = Pearson’s correlation; *p* = p-value; α = alpha for comparison; BF = Bayes factor; IFG = inferior frontal gyrus; pre-SMA = pre-supplementary motor area; STR = striatum; GPe = globus pallidus externa; GPi = globus pallidus interna; Atlas STN = subthalamic nucleus defined by the ATAG atlas; Atlas SN =substantia nigra defined by the ATAG atlas; THAL = thalamus; Manual STN = subthalamic nucleus defined manually; Manual SN = substantia nigra defined by the ATAG atlas but with voxels identified as STN (manually) excluded. No *p*<0.05 or BFs>3 were found. All degrees of freedom=28.

## Relationship between stop signal delay and %BOLD

Correlation analyses were conducted between stop signal delay (SSD) and %BOLD extracted from cortical and subcortical ROIs from stop signal > stop no-signal. Within a single GLM, a stop signal > stop no-signal contrast was computed for each of the 6 SSDs used per participant. Note that SSDs were labelled 1-6 from shortest to longest SSD, irrespective of the exact SSD used because of the variation in SSDs across fMRI runs. As within-subject data were highly correlated, separate analyses were conducted for each participant and each ROI separately. Resultant coefficients were subject to frequentist and Bayesian one sample t-tests (SI table 11).

Paired sample t-tests were also conducted to compare activity below and above the 50%SSD. %BOLD was extracted for ROIs from the contrast of stop signal trials presented before the 50%SSD > stop no-signal and the contrast of stop signal trials presented after the 50%SSD > stop no-signal, and compared (SI table 11). This was used to establish if there were differences in activity prior to, and after, signal onset in the SST.

Differences in activity in ROIs crucial to response inhibition were expected as a function of SSD. This is because the longer the SSD, the more difficult it is to stop a response, and so greater regional activation may be required:

- Evidence for a relationship between %BOLD and SSD was found in bilateral pre-SMA, bilateral THAL (SI table 11) and left SN (defined using the ATAG atlas and manually), although analyses did not survive correction for multiple comparisons. These regions were recruited to a greater extent as SSD increased.
- Although they did not survive correction for multiple comparisons, significant changes in activity were identified in right pre-SMA and bilateral THAL when signals were presented after the 50%SSD (i.e. long SSDs where there was a low probability of stop–signal success) relative to when signals were presented prior to the 50%SSD (i.e. short SSDs where there was a high probability of stop signal success). This may be due to the increased effort in cancelling a response or increased attentional processes associated with performance or error monitoring when stop signal success is limited.

|  |  | Correlation SSD | | | |  | Before / after 50%SSD | | | |
| --- | --- | --- | --- | --- | --- | --- | --- | --- | --- | --- |
| Hem | ROI | t | *p* | α | BF |  | t | *p* | α | BF |
| Right | IFG | 1.17 | 0.252 |  | 0.36 |  | -0.96 | 0.344 |  | 0.30 |
|  | pre-SMA | 2.54 | 0.017 |  | 2.90 |  | -2.51 | 0.018 |  | 2.74 |
|  | STR | 1.10 | 0.282 |  | 0.34 |  | -0.28 | 0.783 |  | 0.20 |
|  | GPe | 1.42 | 0.167 |  | 0.48 |  | -1.43 | 0.164 |  | 0.49 |
|  | GPi | 0.77 | 0.450 |  | 0.26 |  | -0.73 | 0.469 |  | 0.25 |
|  | Atlas STN | 0.54 | 0.592 |  | 0.22 |  | -1.05 | 0.304 |  | 0.32 |
|  | Atlas SN | 1.66 | 0.108 |  | 0.66 |  | -1.36 | 0.185 |  | 0.45 |
|  | THAL | 2.53 | 0.017 |  | 2.88 |  | -2.25 | 0.032 |  | 1.70 |
|  | Manual STN | -0.59 | 0.557 |  | 0.23 |  | 0.02 | 0.988 |  | 0.19 |
|  | Manual SN | 1.67 | 0.107 |  | 0.67 |  | -1.31 | 0.201 |  | 0.42 |
| Left | IFG | 0.38 | 0.705 |  | 0.21 |  | 0.25 | 0.805 |  | 0.20 |
|  | pre-SMA | 2.21 | 0.035 |  | 1.59 |  | -1.49 | 0.146 |  | 0.53 |
|  | STR | 0.86 | 0.395 |  | 0.27 |  | -0.46 | 0.647 |  | 0.22 |
|  | GPe | 1.65 | 0.110 |  | 0.65 |  | -1.65 | 0.109 |  | 0.65 |
|  | GPi | 0.63 | 0.532 |  | 0.23 |  | -1.99 | 0.056 |  | 1.09 |
|  | Atlas STN | 1.80 | 0.083 |  | 0.80 |  | -1.29 | 0.209 |  | 0.41 |
|  | Atlas SN | 2.07 | 0.048 |  | 1.24 |  | -1.92 | 0.065 |  | 0.98 |
|  | THAL | 2.96 | 0.006 | 0.0025 | **6.93** |  | -3.20 | 0.003 | 0.0025 | **11.47** |
|  | Manual STN | 1.97 | 0.059 |  | 1.05 |  | -1.83 | 0.078 |  | 0.84 |
|  | Manual SN | 2.08 | 0.046 |  | 1.28 |  | -1.92 | 0.065 |  | 0.97 |

**SI table 11. T-test results exploring the relationship between %BOLD and SSD.**

Results from one-sample t-tests conducted on r values acquired from within-subject correlations between %BOLD and SSD (Correlation SSD) and from paired sample t-tests comparing %BOLD before and after signal onset (50%SSD). Hem= hemisphere; ROI = region of interest; t = t-statistic; *p* = p-value; α = alpha for comparison; BF = Bayes factor; IFG = inferior frontal gyrus; pre-SMA = pre-supplementary motor area; STR = striatum; GPe = globus pallidus externa; GPi = globus pallidus interna; Atlas STN = subthalamic nucleus defined by the ATAG atlas; Atlas SN =substantia nigra defined by the ATAG atlas; THAL = thalamus; Manual STN = subthalamic nucleus defined manually; Manual SN = substantia nigra defined by the ATAG atlas but with voxels identified as STN (manually) excluded. BFs>3 are presented in bold. *p*<0.05 failed to survive correction for multiple comparisons. All degrees of freedom=29 for one-sample t-tests and for paired sample t-tests.

## Difference between %BOLD on successful vs. unsuccessful stop trials

Paired-sample t-tests were conducted to compare activity in cortical and subcortical ROIs on successful vs. unsuccessful stop trials. Within a single GLM contrasts for successful stops > stop no-signal and unsuccessful stops > stop no-signal were computed. Results are summarised in SI table 12.

We expected that those regions crucial for stopping a response would be recruited to a greater extent on successful vs. unsuccessful stops:

- This was evident in bilateral Striatum (STR), which showed significantly greater activity on successful vs. unsuccessful stop-signal trials (SI table 12), indicating this region is important for the implementation of successful inhibition.
- However, activity in the right pre-SMA showed greater activity on unsuccessful vs. successful stop-signal trials (SI table 12), consistent with a possible role in performance or error monitoring proposed above (although note, activity in this region failed to survive correction for multiple comparisons).

| Hem | ROI | t | *p* | α | BF |
| --- | --- | --- | --- | --- | --- |
| Right | IFG | -0.45 | 0.658 |  | 0.21 |
|  | pre-SMA | -2.31 | 0.028 | 0.0028 | **1.89** |
|  | STR | 3.81 | **<0.001** | 0.0026 | **46.92** |
|  | GPe | 0.79 | 0.435 |  | 0.26 |
|  | GPi | -0.4 | 0.694 |  | 0.21 |
|  | Atlas STN | -0.09 | 0.93 |  | 0.2 |
|  | Atlas SN | -0.59 | 0.561 |  | 0.23 |
|  | THAL | -0.79 | 0.438 |  | 0.26 |
|  | Manual STN | 1.01 | 0.319 |  | 0.31 |
|  | Manual SN | -0.62 | 0.54 |  | 0.23 |
| Left | IFG | 0.43 | 0.671 |  | 0.21 |
|  | pre-SMA | -0.77 | 0.447 |  | 0.26 |
|  | STR | 3.85 | **<0.001** | 0.0025 | **52.13** |
|  | GPe | 0.48 | 0.636 |  | 0.22 |
|  | GPi | -1.52 | 0.141 |  | 0.54 |
|  | Atlas STN | -1.41 | 0.17 |  | 0.47 |
|  | Atlas SN | -1.27 | 0.213 |  | 0.41 |
|  | THAL | -1.77 | 0.087 |  | 0.78 |
|  | Manual STN | -1.43 | 0.163 |  | 0.49 |
|  | Manual SN | -1.31 | 0.202 |  | 0.42 |

**SI table 12. Paired sample t-tests of %BOLD on successful vs. unsuccessful stop trials.**

Hem= hemisphere; ROI = region of interest; t = t-statistic; *p* = p-value; α = alpha for comparison; BF = Bayes factor; IFG = inferior frontal gyrus; pre-SMA = pre-supplementary motor area; STR = striatum; GPe = globus pallidus externa; GPi = globus pallidus interna; Atlas STN = subthalamic nucleus defined by the ATAG atlas; Atlas SN =substantia nigra defined by the ATAG atlas; THAL = thalamus; Manual STN = subthalamic nucleus defined manually; Manual SN = substantia nigra defined by the ATAG atlas but with voxels identified as STN (manually) excluded Results in bold represent *p*-values that survive correction for multiple comparisons and BFs > 3. All degrees of freedom=29.

## Double-response task analyses

## Correlation between %BOLD and DRT2

Correlation analyses were conducted between DRT2 and %BOLD extracted from cortical and subcortical ROIs from the contrast double signal > double no-signal (SI table 13).

We expected activity in regions crucial for non-inhibitory action-updating to be negatively correlated with DRT2. This was because it was hypothesised that those with longer DRT2s to be less efficient at updating relative to those with shorter DRT2s, due to greater PRP influence in the latter. However:

- No correlations between %BOLD and DRT2 was identified (SI table 13).
- It is possible that these regions may not host mechanisms that impose structural limitations on decision-making processes in the DT.

| DRT2 | | | | |
| --- | --- | --- | --- | --- |
| Hem | ROI | r | *p* | BF |
| Right | IFG | -0.03 | 0.86 | 0.14 |
|  | pre-SMA | -0.11 | 0.57 | 0.17 |
|  | STR | -0.11 | 0.56 | 0.17 |
|  | GPe | -0.09 | 0.65 | 0.16 |
|  | GPi | 0.09 | 0.63 | 0.16 |
|  | Atlas STN | -0.11 | 0.56 | 0.17 |
|  | Atlas SN | -0.24 | 0.20 | 0.31 |
|  | THAL | -0.14 | 0.46 | 0.18 |
|  | Manual STN | -0.07 | 0.70 | 0.15 |
|  | Manual SN | -0.24 | 0.20 | 0.32 |
| Left | IFG | -0.10 | 0.60 | 0.16 |
|  | pre-SMA | -0.24 | 0.20 | 0.32 |
|  | STR | -0.18 | 0.33 | 0.23 |
|  | GPe | -0.03 | 0.87 | 0.14 |
|  | GPi | -0.04 | 0.84 | 0.14 |
|  | Atlas STN | -0.22 | 0.24 | 0.28 |
|  | Atlas SN | -0.02 | 0.92 | 0.14 |
|  | THAL | -0.11 | 0.56 | 0.17 |
|  | Manual STN | -0.06 | 0.77 | 0.15 |
|  | Manual SN | -0.01 | 0.95 | 0.14 |

**SI table 13. Pearson’s correlation analyses between %BOLD and DRT2.**

Hem= hemisphere; ROI = region of interest; r = Pearson’s correlation; *p* = p-value; α = alpha for comparison; BF = Bayes factor; IFG = inferior frontal gyrus; pre-SMA = pre-supplementary motor area; STR = striatum; GPe = globus pallidus externa; GPi = globus pallidus interna; Atlas STN = subthalamic nucleus defined by the ATAG atlas; Atlas SN =substantia nigra defined by the ATAG atlas; THAL = thalamus; Manual STN = subthalamic nucleus defined manually; Manual SN = substantia nigra defined by the ATAG atlas but with voxels identified as STN (manually) excluded. No *p*<0.05 or BFs>3 were found. All degrees of freedom=28.

## Correlation between %BOLD and the size of the PRP

Correlation analyses were conducted between the size of the PRP and %BOLD extracted from cortical and subcortical ROIs from the contrast double signal > double no-signal (SI table 14).

The size of the PRP theoretically represents the magnitude of slowing caused by the central bottleneck in dual-task situations (see SI section 2.1.3.2). We expected activity in regions crucial for non-inhibitory action-updating to be negatively correlated with the size of the PRP. This was because it was hypothesised that those with larger PRPs to be less efficient at updating relative to those with smaller PRPs due to a greater PRP influence. However:

- No correlations between %BOLD and the size of the PRP was identified (SI table 14).
- It is possible that these regions may not host mechanisms that impose structural limitations on decision-making processes in the DT.

| PRP | | | | |
| --- | --- | --- | --- | --- |
| Hem | ROI | r | *p* | BF |
| Right | IFG | 0.18 | 0.33 | 0.23 |
|  | pre-SMA | 0.28 | 0.14 | 0.42 |
|  | STR | -0.08 | 0.68 | 0.15 |
|  | GPe | 0.00 | 1.00 | 0.14 |
|  | GPi | 0.08 | 0.67 | 0.15 |
|  | Atlas STN | -0.08 | 0.67 | 0.15 |
|  | Atlas SN | -0.09 | 0.64 | 0.16 |
|  | THAL | 0.11 | 0.56 | 0.17 |
|  | Manual STN | -0.20 | 0.28 | 0.25 |
|  | Manual SN | -0.09 | 0.64 | 0.16 |
| Left | IFG | 0.14 | 0.46 | 0.19 |
|  | pre-SMA | 0.05 | 0.81 | 0.15 |
|  | STR | -0.13 | 0.48 | 0.18 |
|  | GPe | 0.03 | 0.86 | 0.14 |
|  | GPi | -0.09 | 0.63 | 0.16 |
|  | Atlas STN | -0.13 | 0.49 | 0.18 |
|  | Atlas SN | 0.00 | 1.00 | 0.14 |
|  | THAL | 0.10 | 0.60 | 0.16 |
|  | Manual STN | 0.05 | 0.81 | 0.15 |
|  | Manual SN | 0.00 | 1.00 | 0.14 |

**SI table 14. Pearson’s correlation analyses between %BOLD and size of the PRP.**

Hem= hemisphere; ROI = region of interest; r = Pearson’s correlation; *p* = p-value; α = alpha for comparison; BF = Bayes factor; IFG = inferior frontal gyrus; pre-SMA = pre-supplementary motor area; STR = striatum; GPe = globus pallidus externa; GPi = globus pallidus interna; Atlas STN = subthalamic nucleus defined by the ATAG atlas; Atlas SN =substantia nigra defined by the ATAG atlas; THAL = thalamus; Manual STN = subthalamic nucleus defined manually; Manual SN = substantia nigra defined by the ATAG atlas but with voxels identified as STN (manually) excluded. No *p*<0.05 or BFs > 3 were found. All degrees of freedom=28.

## Relationship between stimulus onset asynchrony and %BOLD

Correlation analyses were conducted between stimulus onset asynchronies (SOAs) and %BOLD from the contrast double signal > double no-signal. Within a single GLM, a double signal > double no-signal contrast was computed for each of the 6 SOAs used per participant. Note that SOAs were labelled 1-6 from shortest to longest SOA, irrespective of the exact SOA used because of the variation in SOAs across fMRI runs. As within-subject data were highly correlated, separate analyses were conducted for each participant and each ROI separately. Resultant coefficients were subject to frequentist and Bayesian one sample t-tests (SI table 15).

Paired-sample t-tests were also conducted to compare activity pre- and post-bottleneck, with the bottleneck identified using the procedure outlined in <https://osf.io/zqefx/> (section 6.1.). %BOLD was extracted for ROIs from the contrast of double signal trials presented before the bottleneck > double no-signal and the contrast of double signal trials presented after the bottleneck > double no-signal, and compared (SI table 15). This was used to establish if there were differences in activity when the PRP is evident relative to those occurring post-bottleneck in the DT. Note that 1 participant was excluded from this analysis only as no SOAs were presented post-PRP across any behavioural runs of the DT. Further, for another participant, pre- and post-PRP %BOLD was computed over 6 (as opposed to 8) runs due to the lack of SOAs presented post-PRP for 2 runs.

Differences in activity in ROIs crucial to non-inhibitory action-updating were expected as a function of SOA. This is because the longer the SOA, the easier it should be to execute a 2^nd^ response as the bottleneck provided by the PRP is more likely to have passed. However:

- Bilateral pre-SMA displayed greater activity with increasing SOA (SI table 15) and is supported by increased %BOLD during post- vs. pre- bottleneck phase (SI table 15). Although, note that neither of these analyses survived correction for multiple comparisons.
- As errors were infrequent in the DT (SI table 2) it is unlikely that this response is associated with post-error monitoring. However, increased pre-SMA activity may be expressed when detecting and responding to a signal which is unexpected and preparation to update responses is low. This explanation is consistent with the similar findings identified with respect to SSD in the SST noted above (see SI section 2.3.1.2).

| Hem | ROI | Correlation SOA | | | | Pre- / post PRP | | | |
| --- | --- | --- | --- | --- | --- | --- | --- | --- | --- |
|  |  | t | *p* | α | BF | t | *p* | α | BF |
| Right | IFG | 1.07 | 0.295 |  | 0.33 | -1.09 | 0.287 |  | 0.34 |
|  | pre-SMA | 3.26 | 0.003 | 0.0025 | **13.16** | -2.94 | 0.007 | 0.0025 | **6.52** |
|  | STR | 0.49 | 0.628 |  | 0.22 | -1.19 | 0.244 |  | 0.38 |
|  | GPe | 0.71 | 0.486 |  | 0.24 | -1.31 | 0.201 |  | 0.43 |
|  | GPi | -1.3 | 0.206 |  | 0.41 | -0.14 | 0.888 |  | 0.2 |
|  | Atlas STN | 0.45 | 0.656 |  | 0.21 | -1.46 | 0.155 |  | 0.51 |
|  | Atlas SN | 1.03 | 0.31 |  | 0.32 | -1.95 | 0.062 |  | 1.03 |
|  | THAL | 1.69 | 0.101 |  | 0.69 | -1.29 | 0.209 |  | 0.42 |
|  | Manual STN | 0.67 | 0.51 |  | 0.24 | -1.55 | 0.132 |  | 0.58 |
|  | Manual SN | 1.01 | 0.323 |  | 0.31 | -1.94 | 0.063 |  | 1.01 |
| Left | IFG | -1.24 | 0.227 |  | 0.39 | -0.54 | 0.596 |  | 0.23 |
|  | pre-SMA | 2.79 | 0.009 |  | **4.8** | -2.19 | 0.037 |  | 1.55 |
|  | STR | 0.01 | 0.994 |  | 0.19 | -0.87 | 0.391 |  | 0.28 |
|  | GPe | 0.62 | 0.537 |  | 0.23 | -1.31 | 0.2 |  | 0.43 |
|  | GPi | 0.09 | 0.928 |  | 0.2 | -0.7 | 0.488 |  | 0.25 |
|  | Atlas STN | -0.27 | 0.793 |  | 0.2 | -1.05 | 0.301 |  | 0.33 |
|  | Atlas SN | 0.14 | 0.89 |  | 0.2 | -1.21 | 0.237 |  | 0.38 |
|  | THAL | 1.59 | 0.124 |  | 0.6 | -1.84 | 0.076 |  | 0.87 |
|  | Manual STN | -0.82 | 0.422 |  | 0.26 | -0.7 | 0.488 |  | 0.25 |
|  | Manual SN | 0.11 | 0.912 |  | 0.2 | -1.19 | 0.245 |  | 0.37 |

**SI table 15. T-test results exploring the relationship between %BOLD and SOA.**

Results from one-sample t-tests conducted on r values acquired from within-subject correlations between %BOLD and SOA (Correlation SOA) and from paired sample t-tests comparing %BOLD before and after signal onset (50%SSD). Hem= hemisphere; ROI = region of interest; t = t-statistic; *p* = p-value; α = alpha for comparison; BF = Bayes factor; IFG = inferior frontal gyrus; pre-SMA = pre-supplementary motor area; STR = striatum; GPe = globus pallidus externa; GPi = globus pallidus interna; Atlas STN = subthalamic nucleus defined by the ATAG atlas; Atlas SN =substantia nigra defined by the ATAG atlas; THAL = thalamus; Manual STN = subthalamic nucleus defined manually; Manual SN = substantia nigra defined by the ATAG atlas but with voxels identified as STN (manually) excluded. BFs > 3 are presented in bold. All *p*-values <0.05 failed survive correction for multiple comparisons. For correlation SOA analyses, degrees of freedom=29. For pre / post-PRP analyses, degrees of freedom=28.

## Lateralisation analyses

Tests of laterality were applied to pre-specified ROIs (2.3.3.1) and then to whole brain analyses (2.3.3.2).

**2.3.3.1. Lateralisation of ROIs**

Paired sample t-tests were conducted between %BOLD extracted from left and right cortical and subcortical ROIs to establish if there was evidence of lateralisation of inhibitory (stop signal > stop no-signal) and non-inhibitory action-updating (double signal > double no-signal) networks, separately (SI table 16). The results complement those reported in the manuscript.

Based on previous literature we expected right-lateralised activity to be associated with response inhibition, and left-lateralised activity to be associated with non-inhibitory action-updating:

- For cortical sites (IFG and pre-SMA) activity was greater in the right hemisphere for both inhibitory and non-inhibitory action-updating (SI table 16).
- When responses were stopped, activity in subcortical sites was right-lateralised, with the exception of the GPi (although activity in this site failed to reach statistical significance and had an associated BF <1/3).
- When responses were added to, activity in subcortical sites was consistently left-lateralised (with the exception of the STR), although all comparisons failed to reach statistical significance and most (but not all) BFs were <3.

| ROI | Inhibitory action-updating | | | | Non-inhibitory action-updating | | | |  |
| --- | --- | --- | --- | --- | --- | --- | --- | --- | --- |
|  | t | *p* | α | BF | t | *p* | α | BF |  |
| IFG | 7.51 | **<0.001** | 0.005 | **493661.53** | -5.49 | **<0.001** | 0.005 | **3123.00** |  |
| pre-SMA | 6.97 | **<0.001** | 0.0056 | **129823.72** | -4.85 | **<0.001** | 0.0056 | **621.26** |  |
| STR | 4.66 | **<0.001** | 0.0071 | **380.16** | -1.11 | 0.28 |  | 0.34 |  |
| GPe | 1.38 | 0.18 |  | 0.46 | 1.51 | 0.14 |  | 0.54 |  |
| GPi | 0.31 | 0.76 |  | 0.20 | 1.56 | 0.13 |  | 0.57 |  |
| Atlas STN | 2.76 | 0.01 | 0.0083 | 4.50 | 0.59 | 0.56 |  | 0.23 |  |
| Atlas SN | 1.71 | 0.10 |  | 0.71 | 2.51 | 0.02 | 0.0071 | 2.75 |  |
| THAL | 5.50 | **<0.001** | 0.0063 | **3177.41** | 0.92 | 0.36 |  | 0.29 |  |
| Manual STN | -1.99 | 0.06 |  | 1.09 | 2.61 | 0.01 | 0.0063 | 3.37 |  |
| Manual SN | -1.69 | 0.10 |  | 0.69 | 2.49 | 0.02 | 0.0083 | 2.63 |  |

**SI table 16. Paired sample t-tests of %BOLD from right vs. left ROIs under inhibitory and non-inhibitory action-updating conditions.**

ROI = region of interest; t = t-statistic; *p* = p-value; α = alpha for comparison; BF = Bayes factor; IFG = inferior frontal gyrus; pre-SMA = pre-supplementary motor area; STR = striatum; GPe = globus pallidus externa; GPi = globus pallidus interna; Atlas STN = subthalamic nucleus defined by the ATAG atlas; SN =substantia nigra defined by the ATAG atlas; THAL = thalamus; Manual STN = subthalamic nucleus defined manually; Manual SN = substantia nigra defined by the ATAG atlas but with voxels identified as STN (manually) excluded. Positive t-statistics demonstrate right > left activity. Results in bold represent *p*-values that survive correction for multiple comparisons and BFs > 3. All degrees of freedom=29.

**2.3.3.2. Lateralisation of cortical clusters**

Comparisons of laterality were explored on the basis of the numbers of voxels which survived clusters based correction for multiple comparisons (Z>2.3, see section 2.7.2). These were subjected to Bayesian and frequentist repeated measures ANOVA’s with factors of context (SST, DT, IT) and hemisphere (left and right), and follow up paired t-tests applied to each context. These were applied to signal trials in each context to assess the reactive aspects of each context, as well as to non-single trials to assess the more general context specificity of laterality.

For signal trials there was a significant interaction between context and hemisphere (F_(2,58)_=43.53, *p*<0.001, BF=424.60), a main effect of context (F_(2,58)_=29.44, *p*<0.001, BF=4.739×10^+13^) and no main effect of hemisphere across contexts F_(1,29)_=2.50, *p*=0.125, BF=0.28. There were more voxels active in right compared to left hemisphere for the SST (*p*_0.0167_<0.001, BF=5582.14) and conversely more in the left compared to right for the DT (*p*_0.025_<0.020, BF=2.52). While there were numerically more voxels active in the right compared to left in the IT this difference was not significant (*p*_0.05_=0.744, BF=0.20). For non-signal trials the interaction between context and hemisphere was more ambiguous (F_(2,58)_=11.11, *p*<0.001. BF=0.76), there was no main effect of context (F_(2,58)_=1.51, *p*=0.230, BF=0.39), but a main effect of hemisphere (F_(1,29)_=5.29, *p*=0.029, BF=3.58). The paired comparisons between left and right indicated a difference in the IT there were significantly more voxels in the left than right (*p*_0.0167_<0.011, BF=848.16) consistent with the response on non-signal trials being made with the right hand, and similarly but with weaker evidence in the DT (left > right *p*_0.025_=0.039, BF=1.45). An effect which may have been countermanded by the right laterality of the stop context, resulting in non-significant differences in voxel count in non-signal trials of the stop task (SST right >left *p*_0.05_=0.819, BF=0.20).

These exploratory findings are consistent with the conclusions of the main text that response inhibition is right lateralised and action updating appears to be more left lateralised and these effects are most pronounced when reactive response updating takes place.

**2.4. Temporal signal to noise maps**

Figure SI 3 depicts averaged group tSNR maps and table SI 17 reports the tSNR ratio values for each of the main ROI’s specified in the main text. This indicates that while tSNR is lower around the basal ganglia structures in comparison to the higher cortical ROI’s (Brooks et al., 2013) the average over single runs are comparable, to and within the optimal range, of those previously reported (Murphy et al., 2007).


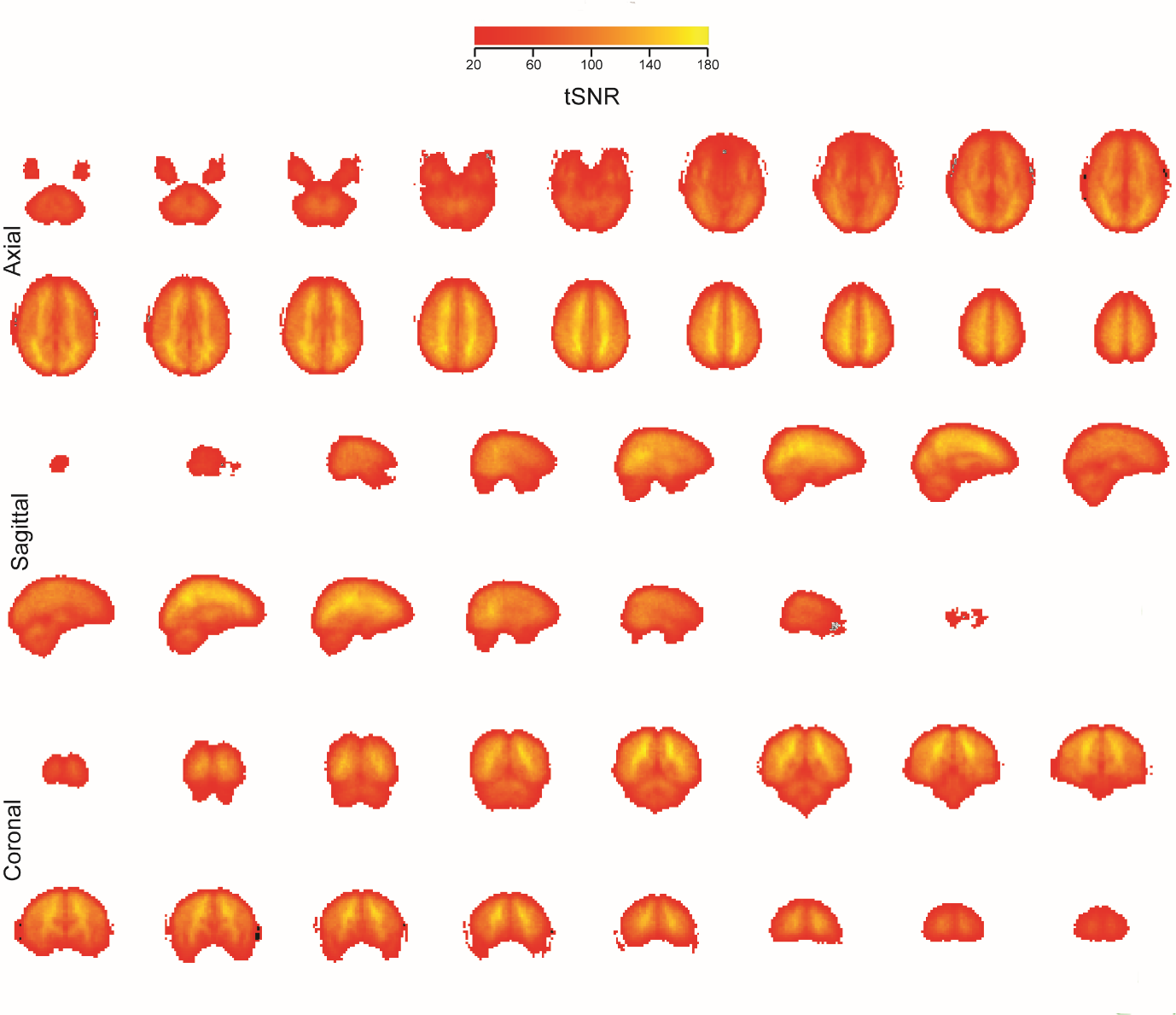


**SI figure 3. Temporal signal to noise (tSNR) maps.**

Illustration of group mean tSNR map applied to participant means across runs. tSNR maps are the result of diving mean by standard deviation across time for each run of data and offer an indication of differences in sensitivity across regions of interest (Murphy et al., 2007).

| ROI | | tSNR | |
| --- | --- | --- | --- |
|  |  | Mean | SD |
| Left | IFG | 122.68 | 31.48 |
|  | Pre-SMA | 123.48 | 30.27 |
|  | STR | 114.54 | 24.50 |
|  | GPe | 108.49 | 26.35 |
|  | GPi | 98.62 | 24.61 |
|  | STN | 77.42 | 19.85 |
|  | SN | 81.22 | 32.36 |
|  | Thal | 87.43 | 19.19 |
| Right | IFG | 94.91 | 30.41 |
|  | Pre-SMA | 132.64 | 33.93 |
|  | STR | 91.35 | 23.40 |
|  | GPe | 82.52 | 24.81 |
|  | GPi | 63.31 | 25.10 |
|  | STN | 70.67 | 19.13 |
|  | SN | 81.22 | 32.36 |
|  | Thal | 70.39 | 18.34 |

**SI table 17. Temporal signal to noise (tSNR) values for regions of interest.**

tSNR values for mean and standard deviation (SD) across participants are provided which are the result of division of mean by standard deviation across the data’s time series. ROI = region of interest; IFG = inferior frontal gyrus; pre-SMA = pre-supplementary motor area; STR = striatum; GPe = globus pallidus externa; GPi = globus pallidus interna; STN = subthalamic nucleus; SN =substantia nigra; Thal = thalamus;

**2.5 Effect size estimates for interrelations between ROIs**

Following recommendations of Baron and Kenny (1986), in addition to the critical statistics reported in table 2 of the manuscript, we report the corresponding summary of effect size estimates as r values in table SI 18.

|  |  |  | Original | Left | | Right | | | | | | |
| --- | --- | --- | --- | --- | --- | --- | --- | --- | --- | --- | --- | --- |
|  |  |  |  | GPe | THAL | pre-SMA | IFG | STR | GPe | SN | STN | THAL |
| Response execution | Left | Gpe | 0.488 |  | 0.113 | 0.582 | 0.652 |  |  |  |  |  |
|  |  | THAL | 0.541 | 0.288 |  | 0.640 | 0.588 |  |  |  |  |  |
|  | Right | pre-SMA | 0.668 | 0.721 | 0.734 |  | 0.134 |  |  |  |  |  |
|  |  | IFG | 0.784 | 0.842 | 0.803 | 0.563 |  |  |  |  |  |  |
| Response inhibition | Right | pre-SMA | 0.778 |  |  |  | 0.201 | 0.545 | 0.703 | 0.664 | 0.664 | 0.627 |
|  |  | IFG | 0.828 |  |  | 0.486 |  | 0.635 | 0.770 | 0.746 | 0.721 | 0.719 |
|  |  | STR | 0.716 |  |  | 0.360 | 0.270 |  | 0.614 | 0.558 | 0.547 | 0.495 |
|  |  | Gpe | 0.484 |  |  | 0.130 | 0.081 | 0.146 |  | 0.225 | 0.226 | 0.071 |
|  |  | SN | 0.543 |  |  | 0.030 | 0.065 | 0.065 | 0.355 |  | 0.121 | 0.162 |
|  |  | STN | 0.589 |  |  | 0.271 | 0.013 | 0.246 | 0.437 | 0.295 |  | 0.337 |
|  |  | THAL | 0.595 |  |  | 0.078 | 0.069 | 0.010 | 0.401 | 0.329 | 0.351 |  |

**Table SI 18. Effect size estimates as r values for the moderator and mediator analyses.**

Presented are the r values for the regressions performed as part of the analyses oriented toward the interrelationships between structures in section 3.3.1.2 of the manuscript and summarised in table 2.

# **References**

Aickin, M., Gensler, H., 1996. Adjusting for multiple testing when reporting research results: the Bonferroni vs Holm methods. Am. J. Public Health 86, 726–728. https://doi.org/10.2105/ajph.86.5.726

Baron, R.M., Kenny, D.A., 1986. The moderator-mediator variable distinction in social psychological research: conceptual, strategic, and statistical considerations. J. Pers. Soc. Psychol. 51, 1173–1182.

Brainard, D.H., 1997. The Psychophysics Toolbox. Spat. Vis. 10, 433–436.

Bright, M.G., Murphy, K., 2013. Removing motion and physiological artifacts from intrinsic BOLD fluctuations using short echo data. Neuroimage 64, 526–37. https://doi.org/10.1016/j.neuroimage.2012.09.043

Brooks, J.C.W., Faull, O.K., Pattinson, K.T.S., Jenkinson, M., 2013. Physiological noise in brainstem FMRI. Front. Hum. Neurosci. 7, 623.

Desikan, R.S., Segonne, F., Fischl, B., Quinn, B.T., Dickerson, B.C., Blacker, D., Buckner, R.L., Dale, A.M., Maguire, R.P., Hyman, B.T., Albert, M.S., Killiany, R.J., 2006. An automated labeling system for subdividing the human cerebral cortex on MRI scans into gyral based regions of interest. Neuroimage 31, 968–980. https://doi.org/S1053-8119(06)00043-7 [pii]10.1016/j.neuroimage.2006.01.021

Frazier, J.A., Chiu, S., Breeze, J.L., Makris, N., Lange, N., Kennedy, D.N., Herbert, M.R., Bent, E.K., Koneru, V.K., Dieterich, M.E., Hodge, S.M., Rauch, S.L., Grant, P.E., Cohen, B.M., Seidman, L.J., Caviness, V.S., Biederman, J., 2005. Structural brain magnetic resonance imaging of limbic and thalamic volumes in pediatric bipolar disorder. Am. J. Psychiatry 162, 1256–1265. https://doi.org/10.1176/appi.ajp.162.7.1256

Goldstein, J.M., Seidman, L.J., Makris, N., Ahern, T., O’Brien, L.M., Caviness Jr, V.S., Kennedy, D.N., Faraone, S. V, Tsuang, M.T., 2007. Hypothalamic abnormalities in schizophrenia: sex effects and genetic vulnerability. Biol. Psychiatry 61, 935–945.

Keuken, M.C., Bazin, P.-L., Crown, L., Hootsmans, J., Laufer, A., Müller-Axt, C., Sier, R., van der Putten, E.J., Schäfer, A., Turner, R., Forstmann, B.U., 2014. Quantifying inter-individual anatomical variability in the subcortex using 7 T structural MRI. Neuroimage 94, 40–6. https://doi.org/10.1016/j.neuroimage.2014.03.032

Logan, G.D., Cowan, W.B., 1984. On the Ability to Inhibit Thought and Action - a Theory of an Act of Control. Psychol. Rev. 91, 295–327. https://doi.org/Doi 10.1037//0033-295x.91.3.295

Makris, N., Goldstein, J.M., Kennedy, D., Hodge, S.M., Caviness, V.S., Faraone, S. V, Tsuang, M.T., Seidman, L.J., 2006. Decreased volume of left and total anterior insular lobule in schizophrenia. Schizophr. Res. 83, 155–171. https://doi.org/10.1016/j.schres.2005.11.020

Murphy, K., Bodurka, J., Bandettini, P.A., 2007. How long to scan? The relationship between fMRI temporal signal to noise ratio and necessary scan duration. Neuroimage 34, 565–574. https://doi.org/10.1016/j.neuroimage.2006.09.032

Pashler, H., 1994. Dual-task interference in simple tasks: data and theory. Psychol. Bull. 116, 220–244.

Pashler, H., Johnston, J.C., 1989. Chronometric evidence for central postponement in temporally overlapping tasks. Q. J. Exp. Psychol. 41, 19–45.

Ruthruff, E., Pashler, H.E., Hazeltine, E., 2003. Dual-task interference with equal task emphasis: Graded capacity sharing or central postponement? Percept. Psychophys. 65, 801–816.

Telford, C.W., 1931. The refractory phase of voluntary and associative responses. J. Exp. Psychol. 14, 1–36. https://doi.org/10.1037/h0073262

Verbruggen, F., Aron, A.R., Band, G.P., Beste, C., Bissett, P.G., Brockett, A.T., Brown, J.W., Chamberlain, S.R., Chambers, C.D., Colonius, H., Colzato, L.S., Corneil, B.D., Coxon, J.P., Dupuis, A., Eagle, D.M., Garavan, H., Greenhouse, I., Heathcote, A., Huster, R.J., Jahfari, S., Kenemans, J.L., Leunissen, I., Li, C.-S.R., Logan, G.D., Matzke, D., Morein-Zamir, S., Murthy, A., Pare, M., Poldrack, R.A., Ridderinkhof, K.R., Robbins, T.W., Roesch, M., Rubia, K., Schachar, R.J., Schall, J.D., Stock, A.-K., Swann, N.C., Thakkar, K.N., van der Molen, M.W., Vermeylen, L., Vink, M., Wessel, J.R., Whelan, R., Zandbelt, B.B., Boehler, C.N., 2019. A consensus guide to capturing the ability to inhibit actions and impulsive behaviors in the stop-signal task. Elife 8. https://doi.org/10.7554/eLife.46323

Verbruggen, F., Aron, A.R., Stevens, M.A., Chambers, C.D., 2010. Theta burst stimulation dissociates attention and action updating in human inferior frontal cortex. PNAS 107, 13966–13971. https://doi.org/10.1073/pnas.1001957107

1. Initial reaction times on double signal trials consistently >500ms. [↑](#footnote-ref-1)
2. 1 participant = mean reaction times on stop no-signal trials >600ms; 1 participant = overall double signal trials accuracy <85%; 1 participant = overall double signal trial accuracy <85% and mean reaction times on double signal trials >500ms. [↑](#footnote-ref-2)
